# Pyruvate dehydrogenase complex—enzyme 2, a new target for *Listeria* spp. detection identified using combined phage display technologies

**DOI:** 10.1038/s41598-020-72159-4

**Published:** 2020-09-17

**Authors:** Gustavo Marçal Schmidt Garcia Moreira, Sarah Mara Stella Köllner, Saskia Helmsing, Lothar Jänsch, Anja Meier, Sabine Gronow, Christian Boedeker, Stefan Dübel, Marcelo Mendonça, Ângela Nunes Moreira, Fabricio Rochedo Conceição, Michael Hust

**Affiliations:** 1 Abteilung Biotechnologie, Institut für Biochemie, Biotechnologie und Bioinformatik, Technische Universität Braunschweig, Braunschweig, Lower Saxony Germany; 2 Cellular Proteomics, Helmholtz Centre for Infection Research, Braunschweig, Lower Saxony Germany; 3 Leibniz Institute DSMZ-German Collection of Microorganisms and Cell Cultures, Braunschweig, Lower Saxony Germany; 4 Departamento de Medicina Veterinária, Universidade Federal do Agreste de Pernambuco, Garanhuns, PE Brazil; 5 Laboratório de Imunologia Aplicada, Núcleo de Biotecnologia, Centro de Desenvolvimento Tecnológico, Universidade Federal de Pelotas, Pelotas, RS Brazil

**Keywords:** Applied microbiology, Infectious-disease diagnostics, Pathogens, Biotechnology, Applied immunology

## Abstract

The genus *Listeria* comprises ubiquitous bacteria, commonly present in foods and food production facilities. In this study, three different phage display technologies were employed to discover targets, and to generate and characterize novel antibodies against *Listeria*: antibody display for biomarker discovery and antibody generation; ORFeome display for target identification; and single-gene display for epitope characterization. With this approach, pyruvate dehydrogenase complex—enzyme 2 (PDC-E2) was defined as a new detection target for *Listeria*, as confirmed by immunomagnetic separation-mass spectrometry (IMS-MS). Immunoblot and fluorescence microscopy showed that this protein is accessible on the bacterial cell surface of living cells. Recombinant PDC-E2 was produced in *E. coli* and used to generate 16 additional antibodies. The resulting set of 20 monoclonal scFv-Fc was tested in indirect ELISA against 17 *Listeria* and 16 non-*Listeria* species. Two of them provided 100% sensitivity (CI 82.35–100.0%) and specificity (CI 78.20–100.0%), confirming PDC-E2 as a suitable target for the detection of *Listeria*. The binding region of 18 of these antibodies was analyzed, revealing that ≈ 90% (16/18) bind to the lipoyl domains (LD) of the target. The novel target PDC-E2 and highly specific antibodies against it offer new opportunities to improve the detection of *Listeria*.

## Introduction

The genus *Listeria* comprises Gram-positive, facultative anaerobe, non-sporulating, rod-shaped bacteria. There were 6 *Listeria* species described until 2009, from which the last species was described in 1984^1^. Later on, 14 new species were described^2–8^ resulting in a total of 20: *L. monocytogenes*, *L. ivanovii*, *L. innocua*, *L. seeligeri*, *L. welshimeri*, *L. grayi*, *L. marthii*, *L. rocourtiae*, *L. fleischmannii*, *L. weihenstephanensis*, *L. booriae*, *L. newyorkensis*, *L. floridensis*, *L. aquatica*, *L. cornellensis*, *L. riparia*, *L. grandensis*, *L. goaenensis*, *L. costaricensis*, and *L. thailandensis*. This intense rate of new species in recent years reflects the increasing perception of the *Listeria* genus as important objects of study. The most relevant pathogen for humans is still *L. monocytogenes*, while *L. ivanovii* is more important for veterinary medicine^9^. Nevertheless, *L. monocytogenes* strains containing genes from *L. ivanovii* have been described to be hypervirulent, augmenting the importance of both species^10^. Currently, 13 different serotypes of *L. monocytogenes* were described, of which three (4b, 1/2a, and 1/2b) are considered most virulent and responsible for 95–98% of the human infections^11^.

The *Listeria* genus is ubiquitous, which can be found in many places in the environment, as well as in the microbiota of animals and humans. In addition, it has elevated resistance to a broad range of pH and temperature, high concentrations of salts, and to a low amount of oxygen, as well as the capacity of forming biofilms. These characteristics contribute to their constant presence in food production facilities^12–14^. This, combined with its sophisticated mechanism for gut invasion, makes *L. monocytogenes* a significant foodborne pathogen. The disease caused by these bacteria is called listeriosis and affects mostly immunocompromised people, such as transplanted, cancer, and HIV patients, as well as infants, elderly, pregnant women, and their fetuses. Even though having a low incidence (less than 1/100,000 people in most of the countries), listeriosis is a serious threat since the mortality rate is around 25% worldwide^15^. Because of this, in countries such as the USA, laws requiring “zero tolerance” of *Listeria* in ready-to-eat (RTE) foods are applied^14^. In contrast, in many parts of Europe, the laws are less strict, allowing up to 100 CFU/g of in RTE foods^16^. In other countries, there is either fewer or no legislation in respect of certain high-risk foods, making *Listeria* contamination an even bigger concern. Therefore, the detection of *Listeria* in produced food or food production facilities is essential. The standard method for its detection in food involves lengthy microbiological procedures for enrichment, isolation, and biochemical characterization^17^. Although being very specific and precise, the whole procedure takes around 7 days from sample collection to the result. As an alternative, procedures such as PCR-based techniques, immunological methods, proteomic approaches, and different formats of biosensors have been developed in order to reduce the detection time to around 1 day or less^18^.

Among these technologies, antibody-based assays, such as lateral flow tests, are considered the most promising, since they offer a simple, quick, and low-cost detection. Although very attractive for the food industry, lateral flow assays, as well as other new methods, still depend on an efficient enrichment step prior to detection to increase the number of detectable cells in the sample. Significant improvement in the enrichment step has been achieved, dramatically reducing the time to obtain detectable amounts of bacterial cells^19^. In another study, it was possible to successfully enhance the limit of detection of the lateral flow through the improvement of colorimetric components^20^. However, the development of appropriate and specific monoclonal antibodies (mAbs) for the lateral flow assay did not advance in the same way, leaving room for improvement.

Most of the studied detection targets for *Listeria* are related to the detection of *L. monocytogenes*, such as the internalins A (InlA) and B (InlB)^21,22^. These proteins are involved in the pathogenesis during the intestinal invasion step and are accessible on the cell surface through attachment to the cell wall. In addition to these two proteins, others were also used for the detection of pathogenic species, such as actin polymerization protein (ActA)^23^, or *N*-acetyl muramidase^24,25^. Not only is the detection of pathogenic species important, but also the recognition of non-pathogenic *Listeria*, since it has been shown that non-pathogenic species are able to overgrow the pathogenic during the enrichment step, thus increasing the chance of producing false-negative results in detection^26–28^. In this respect, several targets, such as p60^29^, flagellin^30^, or 1,6-fructose bisphosphate aldolase (FBA)^31^, have been described for the detection of both pathogenic and non-pathogenic *Listeria* species.

Phage display is a versatile technique that has been widely used for the study of antibodies and antigens. As a display method, this approach connects genotype and phenotype, making it easier to access the genetic information of molecules that are selected from an interaction procedure^32^. Through an approach called panning (which refers to “gold panning”), it is possible to select molecules with desired interaction properties. Recombinant mAbs generated by phage display are currently a valuable resource for basic research, diagnostics, and therapeutic applications^33–37^. On the other hand, improvements in antigen phage display technology have been achieved due to the availability of Hyperphage^38^, such as ORFeome phage display^39–41^. This technology can be employed for the discovery of biomarkers, which then can be used for the development of vaccines and diagnostics. Finally, the single-gene phage display can be used to characterize antigen regions where antibodies bind to by identifying the “minimal sequence of recognition” (MSR), which represents the shortest amino acid segment of the antigen that is present in every output sequence of the procedure after alignment^42–44^.

The present study demonstrated that phage display technologies used in combination allowed obtaining a biomarker for detection, antibodies against it, and relevant epitope information. In this regard, novel recombinant monoclonal antibodies were generated against and then used to characterize the newly identified target dihydrolipoamide acetyltransferase (pyruvate dehydrogenase complex—enzyme 2, PDC-E2). In addition, these antibodies allowed the specific detection of all the 17 *Listeria* species included in the study.

## Results

### Antibody panning on *Listeria* protein fractions provided binders for living cells detection

**Figure 1:**
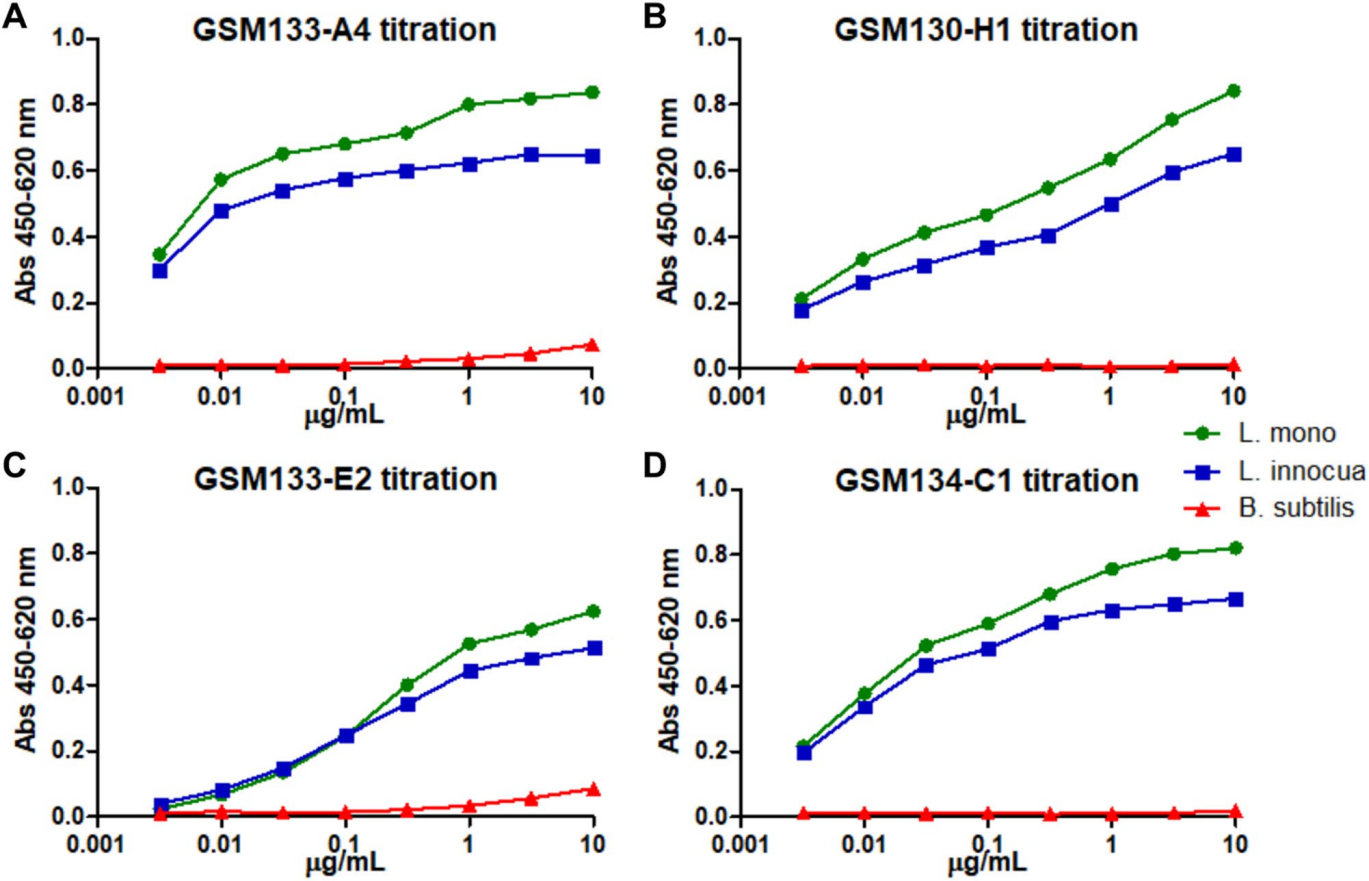
ELISA of the four scFv-Fc antibodies initially generated against *Listeria* protein fractions. The monoclonal scFv-Fc GSM133-A4 (**A**), GSM130-H1 (**B**), GSM133-E2 (**C**), and GSM134-C1 (**D**) were tested against three strains coated alive onto ELISA plates: *L. monocytogenes* ATCC 7644 (L. mono), *L. innocua* DSM 20649 (L. innocua), and *B. subtilis* 168 NCIB 10106 (B. subtilis).

The use of subcellular protein fractions of the cell wall, membrane, and cytoplasm of *Listeria* was performed in order to avoid using whole cells since they tend to detach from the polystyrene surface during panning procedure. In addition, it increases the chances of getting antibodies against exposed proteins, mainly from cell wall and membrane fractions. This way, antibodies in scFv format against *Listeria* protein fractions were generated by phage display (Figure S1) and converted into the scFv-Fc format (with mouse IgG2a Fc). Four of the resulting antibodies showed concentration-dependent binding on living bacterial cells and allowed the detection of both pathogenic (*L. monocytogenes*) and non-pathogenic (*L. innocua*) species of *Listeria* while showing no significant reaction with *B. subtilis* used as negative control (Fig. 1). Although the EC_50_ could not be determined precisely, GSM133-A4 showed the best binding in a dilution ranking, so this antibody was chosen for further initial characterization of the target.

### Target identification by either mass spectrometry or ORFeome phage display provide similar results

**Figure 2:**
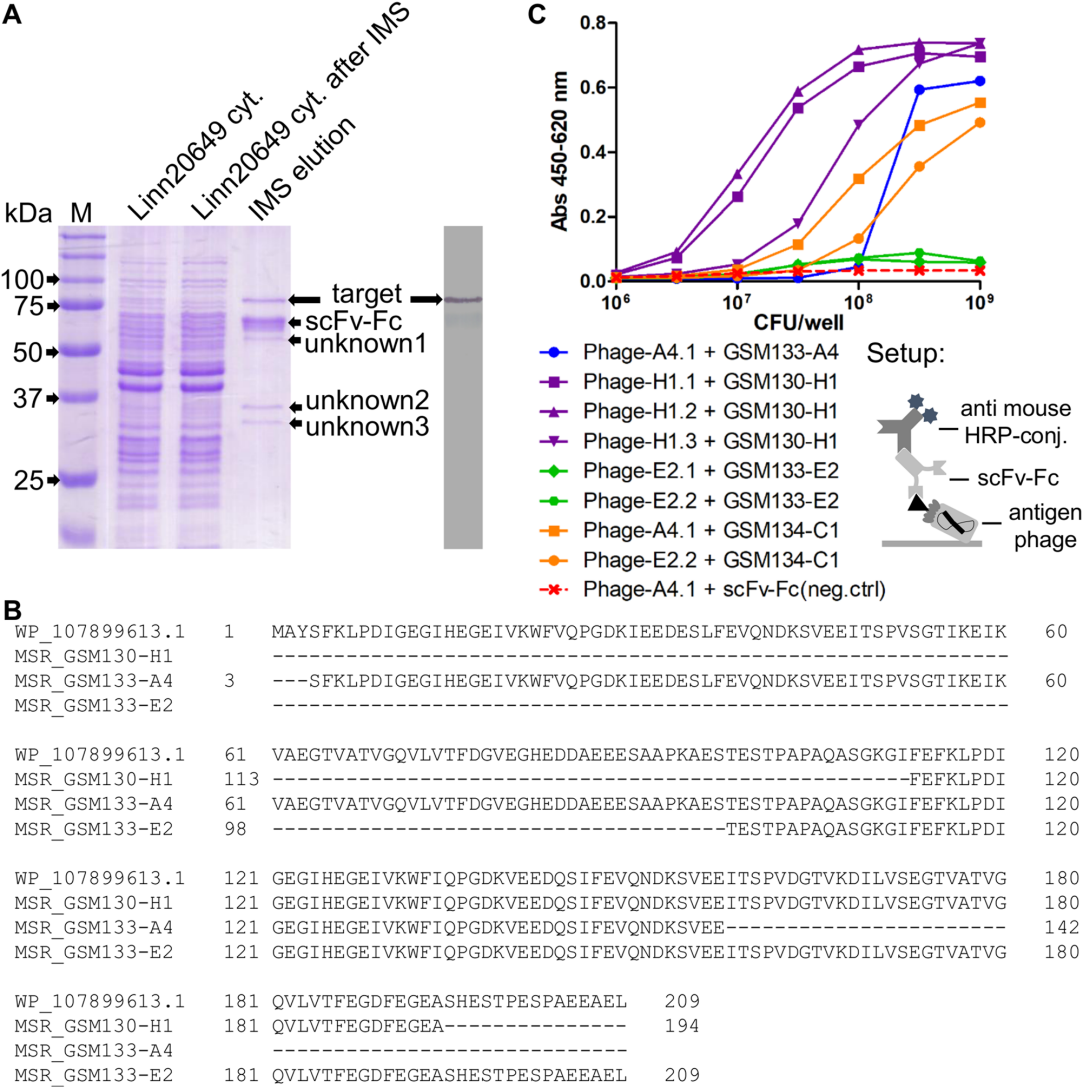
Identification of the target via MS and ORFeome phage display. (**A**) SDS-PAGE and immunoblot after IMS with GSM133-A4 scFv-Fc; M is the protein ladder Precision Plus Protein Unstained (Bio-Rad). (**B**) Alignment of the first 209 amino acids of a reference PDC-E2 sequence (WP_107899613.1) with the MSR of scFv-Fc GSM130-H1, GSM133-A4, and GSM133-E2 after ORFeome phage display. (**C**) Titration curves of the 4 initial scFv-Fc [GSM133-A4 (blue), GSM130-H1 (purple), GSM133-E2 (green), and GSM134-C1 (yellow)] against different amounts of monoclonal phage isolated in the ORFeome phage display for target identification. The setup of the assay is represented below the graphic. The complete SDS-PAGE and immunoblot images can be found, respectively, as Supplementary Figs S7 and S8.

After generating antibodies against protein fractions of *Listeria*, their targets were further identified by IMS-MS (immunomagnetic separation-mass spectrometry) or ORFeome phage display. Prior to the MS analysis, samples for IMS with protein A beads were prepared using GSM133-A4 to isolate the target from the cytoplasmic protein mixture. This procedure led to the proper enrichment of the target, as confirmed by SDS-PAGE and immunoblot (Fig. 2A). In addition to the target, three other protein bands (named as “unknown” 1–3) were also detected in SDS-PAGE, but not in immunoblot. All proteins were cut from the gel and analyzed by MS (Table S1), which identified the target as dihydrolipoamide acetyltransferase (GenBank: WP_010990728.1), also known as pyruvate dehydrogenase complex—enzyme 2 (PDC-E2). The “unknown” proteins 2 and 3 were detected as PDC-E1α (GenBank: WP_072572643.1) and PDC-E1β (GenBank: WP_038409535.1), respectively. This information indicates that the “unknown 1” protein may refer to PDC-E3, but it was not properly analyzable due to its close proximity to the scFv-Fc.

In parallel to the MS analysis, ORFeome phage display was performed to identify the target of the four initial antibodies (GSM130-H1, GSM133-A4, GSM133-E2, and GSM134-C1) using an antigen library built with the genome of *L. monocytogenes* ATCC 7644. With this approach, the identified target was also PDC-E2 (GenBank: WP_107899613.1) (Fig. 2B, Table S1). Out of the four antibodies used, only GSM134-C1 did not allow to identify the target. Moreover, the panning with GSM133-A4 resulted in two hits: one referring to PDC-E2, and another to a non-related protein that was discarded due to low reactivity (data not shown).

To confirm specific binding, a subset of PDC-E2 peptide phage was produced and tested in ELISA on the respective antibodies. All antibody-peptide phage combination showed higher reactions compared to the negative control in a concentration-dependent manner (Fig. 2C). In addition, since the isolated fragments of the 3 antibodies are closely related to each other in protein sequence, some were tested with GSM134-C1, which did not select any fragment on panning. The result showed that this scFv-Fc can recognize the peptides isolated from panning with other antibodies, indicating that it may bind to a similar epitope region. Furthermore, it is important to mention that the reactions of GSM133-E2 were much lower than that of other antibodies, indicating a different behavior of this scFv-Fc.

### PDC-E2 is present in the *Listeria* cell wall protein fraction and is accessible on its surface

**Figure 3:**
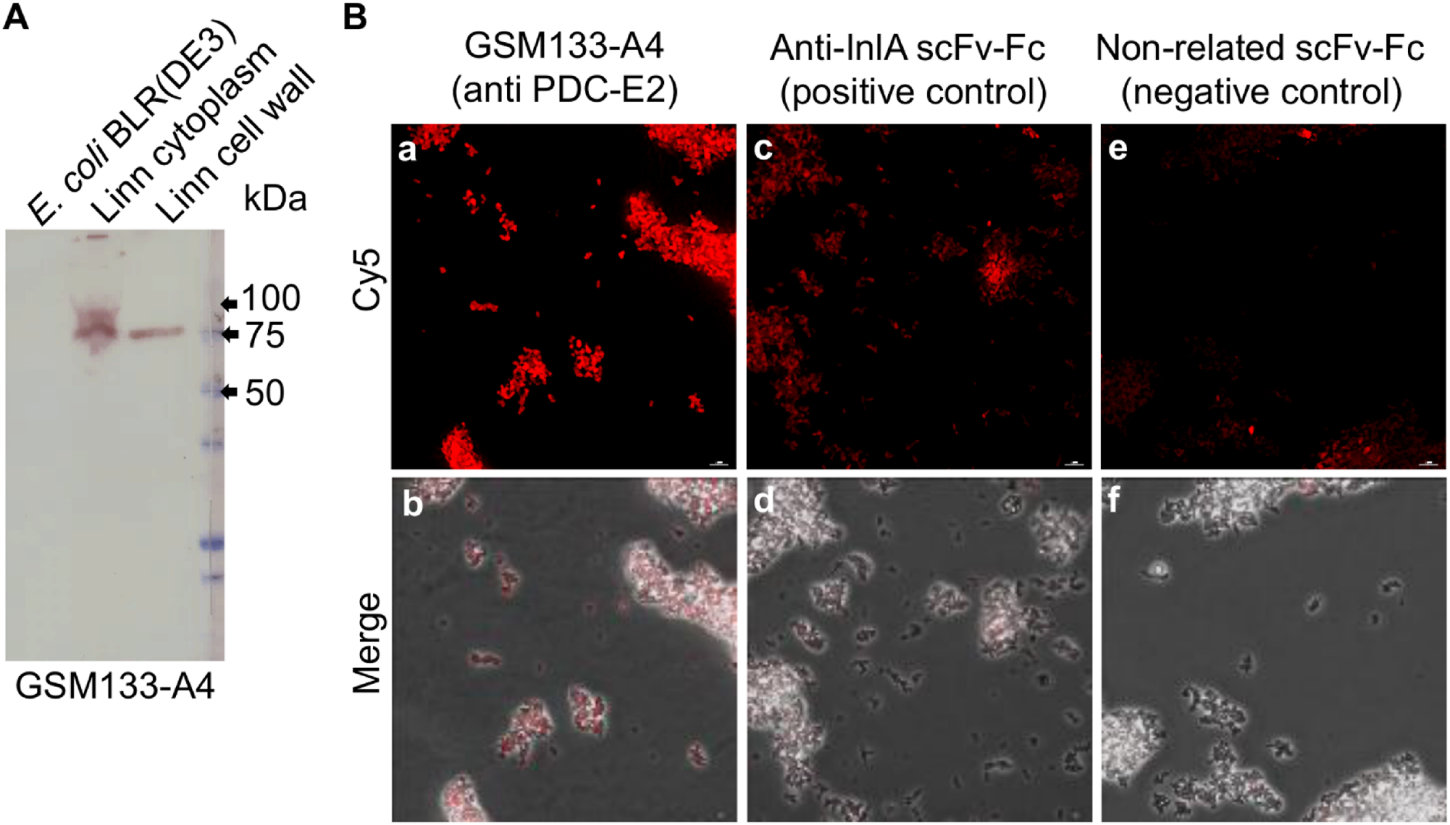
Test of GSM133-A4 in immunoblot of *Listeria* protein fractions and immunofluorescence microscopy. (**A**) Immunoblot of GSM133-A4 of protein fractions from the cytoplasm and cell wall of *L. innocua* DSM20649 (Linn), as well as *E. coli* BLR (DE3) extract used as a negative control. The blot shows that GSM133-A4 antibody binds to a target contained both in the cytoplasm and cell wall fractions of *Listeria*. The used protein ladder was Precision Plus Protein All Blue (Bio-Rad). (**B**) Fluorescence microscopy on alive *L. monocytogenes* 4b DSM 15675. The signal of GSM133-A4 (c and f) showed to be higher than both the negative control with a non-related antibody (a and d) and the anti-InlA positive control (b and e). The complete immunoblot image can be found as Supplementary Fig S9.

To analyze the occurrence of the identified target PDC-E2, which is a protein expected to be located in the cytoplasm, in different *Listeria* cell compartments, antibody GSM133-A4 was used on immunoblot of *Listeria* cytoplasmic and cell wall protein fractions (Fig. 3A). The results indicate that the target antigen is present in the cytoplasm, as well as on the cell wall of *Listeria* cells. This is in accordance with the detection of GSM133-A4 in whole-cell ELISA.

In order to further confirm the accessibility of PDC-E2 on the bacterial surface, GSM133-A4 was employed to analyze *L. monocytogenes* ser. 4b DSM 15675 by immunofluorescence microscopy (Fig. 3B,a,b). As a negative control, a non-related scFv-Fc was used to verify the unspecific binding of the secondary antibody. While the negative control showed imperceptible fluorescence signal (Fig. 3B,c,d), the positive control using an scFv-Fc anti-InlA antibody recognized its well-characterized target linked to the cell wall on the surface of *L. monocytogenes* (Fig. 3B,e,f). In comparison, GSM133-A4 staining showed an even higher signal, demonstrating the accessibility of the target on the *Listeria* cell surface.

### Antibody panning on recombinant PDC-E2 increased the number of binders for *Listeria* spp. detection

In order to increase the set of antibodies recognizing PDC-E2, another antibody panning was done on the purified recombinant antigen. PDC-E2 gene was cloned and expressed as a recombinant protein in *E. coli*. After purification, it was used to select antibodies from HAL9 and HAL10 libraries, which were mixed in the same panning well. With this approach, 16 novel binders were identified and produced as scFv-Fc with a murine Fc part. Initial tests showed that they are all able to recognize recombinant PDC-E2 in immunoblot and bind specifically to *Listeria* spp. in ELISA (Figures S2 and S3).

### PDC-E2 allows the detection of *Listeria* spp. via indirect ELISA

In total, 20 monoclonal scFv-Fc antibodies were tested for *Listeria* spp. detection in indirect ELISA. After performing ROC (Receiver Operating Characteristic) analysis, concentrations showing the best AUC (Area Under Curve) were used to estimate the best equilibrium of sensitivity and specificity. Two scFv-Fc (GSM313-E9, and GSM313-H8) allowed to discriminate *Listeria* spp. from all other tested species with 100% sensitivity and specificity, with AUC = 1.0 (Table 1). The three antibodies with the next best performance are GSM313-F5, GSM313-G12, and GSM130-H1, of which GSM130-H1 is the only one derived from the initial panning on *Listeria* protein fractions.

**Table 1:** Diagnostic performance of all the scFv-Fc targeting PDC-E2.

Antibody	Best concentration^a^	Sensitivity % (CI)	Specificity % (CI)	AUC (CI)
GSM313-E9	EC_50_+	100.0 (82.35–100.0)	100.0 (78.20–100.0)	1.0 (1.0)
GSM313-H8	EC_50_+	100.0 (82.35–100.0)	100.0 (78.20–100.0)	1.0 (1.0)
GSM313-F5	EC_50_+	94.74 (73.97–99.87)	100.0 (78.20–100.0)	0.993 (0.9746–1.011)
GSM313-G5	EC_50_−	100.0 (82.35–100.0)	93.33 (68.05–99.83)	0.993 (0.9745–1.011)
GSM130-H1	EC_50_+	94.74 (73.97–99.87)	93.33 (68.05–99.83)	0.9895 (0.9663–1.013)
GSM313-G12	EC_50_+	89.47 (66.86–98.70)	100.0 (78.20–100.0)	0.986 (0.9574–1.014)
GSM313-D10	EC_50_+	94.74 (73.97–99.87)	93.33 (68.05–99.83)	0.9789 (0.939–1.019)
GSM313-C9	EC_50_+	89.47 (66.86–98.70)	100.0 (78.20–100.0)	0.9579 (0.8904–1.025)
GSM313-D6	EC_50_	84.21 (60.42–96.62)	100.0 (78.20–100.0)	0.9439 (0.8705–1.017)
GSM313-G11	EC_50_+	89.47 (66.86–98.70)	86.67 (59.54–98.34)	0.9439 (0.8693–1.018)
GSM313-B1	EC_50_−	100.0 (82.35–100.0)	80.0 (51.91–95.67)	0.9404 (0.8646–1.016)
GSM134-C1	EC_50_−	94.74 (73.97–99.87)	80.0 (51.91–95.67)	0.8965 (0.7803–1.013)
GSM133-A4	EC_50_	94.74 (73.97–99.87)	80.0 (51.91–95.67)	0.8947 (0.7815–1.008)
GSM313-H11	EC_50_+	89.47 (66.86–98.70)	86.67 (59.54–98.34)	0.8596 (0.7135–1.006)
GSM313-E1	EC_50_+	94.74 (73.97–99.87)	80.0 (51.91–95.67)	0.8246 (0.6465–1.003)
GSM313-F7	EC_50_	73.68 (48.80–90.85)	73.33 (44.90–92.21)	0.7895 (0.618–0.9609)
GSM133-E2	EC_50_−	78.95 (54.43–93.95)	73.33 (44.90–92.21)	0.7789 (0.5889–0.969)
GSM313-B12	EC_50_+	73.68 (48.80–90.85)	66.67 (38.38–88.18)	0.7789 (0.6205–0.9374)
GSM313-F10	EC_50_+	94.74 (73.97–99.87)	66.67 (38.38–88.18)	0.7158 (0.5034–0.9282)
GSM313-G10	EC_50_−	63.16 (38.36–83.71)	66.67 (38.38–88.18)	0.6632 (0.4727–0.8536)

*CI* confidence interval.

^a^The antibody concentrations used were: the EC_50_; a √10-fold concentration above the EC_50_ (EC_50_+); and a √10-fold concentration below (EC_50_−).

**Figure 4:**
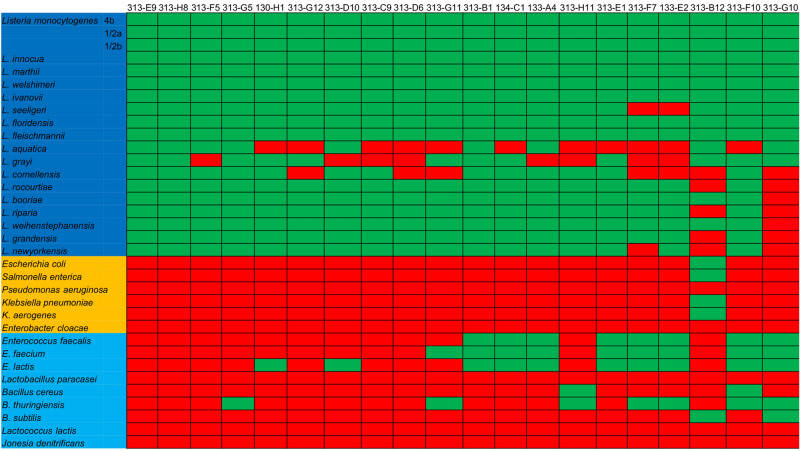
Reaction profile of the 20 antibodies targeting PDC-E2 in indirect ELISA. Indirect ELISA was done with 19 *Listeria* strains (dark blue background) and 15 non-*Listeria* strains, including Gram-negative (golden background) and Gram-positive (light blue background). After performing a ROC curve analysis, the cut-off value for each antibody was used to determine if it was considered to provide positive (green) or negative (red) reactions.

When analyzing the reaction against the tested strains, some significant patterns of recognition were observed. First, the species *L. aquatica*, *L. grayi*, and *L. cornellensis* were difficult to be detected, since out of the 20 antibodies, 11, 8, and 7, respectively, were not able to recognize them (Fig. 4). Another observation is that most of the reactions with non-*Listeria* species were against *Enterococcus* (11/20) and *Bacillus* (8/20) genus, sometimes with more than one species of each genus. Interestingly, PDC-E2 sequences from both *Enterococcus* and *Bacillus* are the ones showing to be closer related to the one from *L. monocytogenes*, having around 50–60% identity, while the other species, except *L. paracasei* (55.7%), did not surpass 45% (Table S2).

### Most of the generated antibodies bind to the lipoyl domains of PDC-E2

To analyze the binding characteristics of the anti-PDC-E2 antibodies in further detail, their MSR (minimal sequence of recognition) was determined by single-gene phage display using a library displaying random fragments of PDC-E2. It was possible to define an MSR for 18 of the 20 antibodies. Interestingly, all but one of the mapped antibodies were shown to bind the same region, which was either the 1st or 2nd lipoyl domain (LD) (Table 2). Interestingly, 4 out of 18 antibodies (GSM133-A4, GSM313-D10, GSM313-F7, and GSM313-G10) were able to recognize both LD domains, although one is always preferred. GSM133-E2, which initially showed very low reactions (Fig. 2C), showed to bind the catalytic domain (MSR that was not initially defined with ORFeome display) and one nonsense peptide (translated peptide does not correspond to PDC-E2). Thus, this antibody was tested in IgG format, which was then able to reproduce the results from ORFeome phage display, although other binding regions that are, in principle, not related to each other were also detected. The last and most intriguing observation of the mapped MSR is regarding GSM313-E9 and GSM313-H8, which were the only antibodies showing 100% sensitivity and specificity in the indirect ELISA experiments and also the only ones to bind a sequence composed by parts from both the 1st and 2nd LD.

**Table 2:** Epitope mapping results of the antibodies used in indirect ELISA.

Antibody	AUC	MSR location (amino acid position)	MSR length (amino acids)	Domain (nº of sequences)
GSM313-E9	1.0	115–185	71	2nd (12/13), 1st + 2nd LD (1/13)
GSM313-H8	1.0	104–188	85	2nd (10/12), 1st + 2nd LD (2/12)
GSM313-F5	0.993	115–185	68	2nd LD (14/14)
GSM313-G5	0.993	103–188	86	2nd LD (11/11)
GSM130-H1	0.9895	113–188	76	2nd LD (23/23)
GSM313-G12	0.986	108–187	80	2nd LD (11/11)
GSM313-D10	0.9789	5–79, 115–188	74, 74	1st (12/15), 2nd (3/15) LD
GSM313-C9	0.9579	5–79	74	1st LD (13/13)
GSM313-D6	0.9439	109–191	83	2nd LD (4/4)
GSM313-G11	0.9439	108–188	81	2nd LD (11/11)
GSM313-B1	0.9404	108–188	81	2nd LD (11/11)
GSM134-C1	0.8965	106–192	87	2nd LD (14/14)
GSM133-A4	0.8947	108–188	81	1st LD (3/21), 2nd LD (18/21)
GSM313-H11	0.8596	232–305	73	PDC-E3 binding domain (12/12)
GSM313-E1	0.8246	NM	NM	NM
GSM313-F7	0.7895	3–77, 115–187	73, 75	1st (9/11), 2nd (2/11) LD
GSM133-E2	0.7789	402–424	23	Catalytic domain (1/2), nonsense (1/2)
GSM133-E2 (IgG)	NT	113–145, 301–359, 526–543	32, 58, 18	2nd LD (9/11), catalytic domain (2/11, different parts)
GSM313-B12	0.7789	5–81	77	1st LD (14/14)
GSM313-F10	0.7158	NM	NM	NM
GSM313-G10	0.6632	3–78, 113–191	79, 76	1st (6/11), 2nd (5/11) LD

*NM* not mapped, *NT* not tested, *LD* lipoyl domain.

The size of the isolated fragments and, thus, the detected MSR of the 4 antibodies used for ORFeome phage display could be reduced when using single-gene phage display (Table 3, see also Texts S1 and S2). The scFv-Fc antibodies GSM130-H1 and GSM133-A4 in single-gene phage display showed MSR that was 8.54 and 48.70% smaller, respectively. While the IgG GSM133-E2 showed a 71.43% reduction in MSR size. In the case of GSM134-C1, only single-gene phage display resulted in reactive sequences allowing to determine the MSR.

**Table 3:** Comparison of the MSR length between ORFeome and single gene phage displays.

Antibody	MSR length (amino acids)		MSR reduction (%) ^a^
	ORFeome display	Single gene phage display	
GSM130-H1	82	75	8.54
GSM133-A4	154	79, 88	48.70, 42.86
GSM133-E2	112	32^b^	71.43
GSM134-C1	No hits	87	–

^a^The MSR reduction indicates how much the fragments from single gene phage display are smaller in comparison to those from ORFeome phage display.

^b^This sequence refers to the MSR in the 2nd LD domain of the IgG molecule, since GSM133-E2 showed multiple MSR.

## Discussion

Phage display is mainly applied to generate antibodies for therapy and research^45^. Nonetheless, this technology was originally conceived for epitope detection^46^. Consequently, antigen phage display methods are still powerful to find biomarkers for diagnostics or vaccine development^47^. The present study uses a combination of three different phage display techniques. Initially, antibody phage display was used for the discovery of new biomarkers from subcellular fractions of *L. monocytogenes*. The generation of antibodies against such complex protein mixtures has also been demonstrated for viruses, such as Porcine Epidemic Diarrhea Virus^48^ and Yellow Fever^49^, and the Gram-negative bacterium *Legionella pneumophila*^50^. However, in the case of viruses, the targets are usually known and their number is diminished compared to subcellular fractions. In the case of *L. pneumophila*, the cells used for panning were previously treated with formaldehyde, which may explain the enrichment of antibodies against lipopolysaccharide (LPS). A similar study employed antibody phage display to generate scFv specifically against *L. monocytogenes* cells^51^. Intriguingly, this latter study employs two different panning strategies, from which all positive hits resulted in the same antibody sequence and, thus, the same target. The present study corroborates this result since four different panning strategies were performed, from which three resulted in useful antibodies against the same target. Although the four initial antibodies generated here represent a higher number compared to the single one described in the previously published article, the fact that different strategies can lead to the same target is an interesting point for further investigation.

In fact, most of the studies on antibody generation via phage display describe the use of purified antigens, suggesting that this approach may be more successful in providing high-quality antibodies^52–56^. Accordingly, in the present study, 16 novel antibodies were generated with this strategy, of which four showed better diagnostic performance than the best of the initial four antibodies generated against cell fractions (see Table 3).

**Figure 5:**
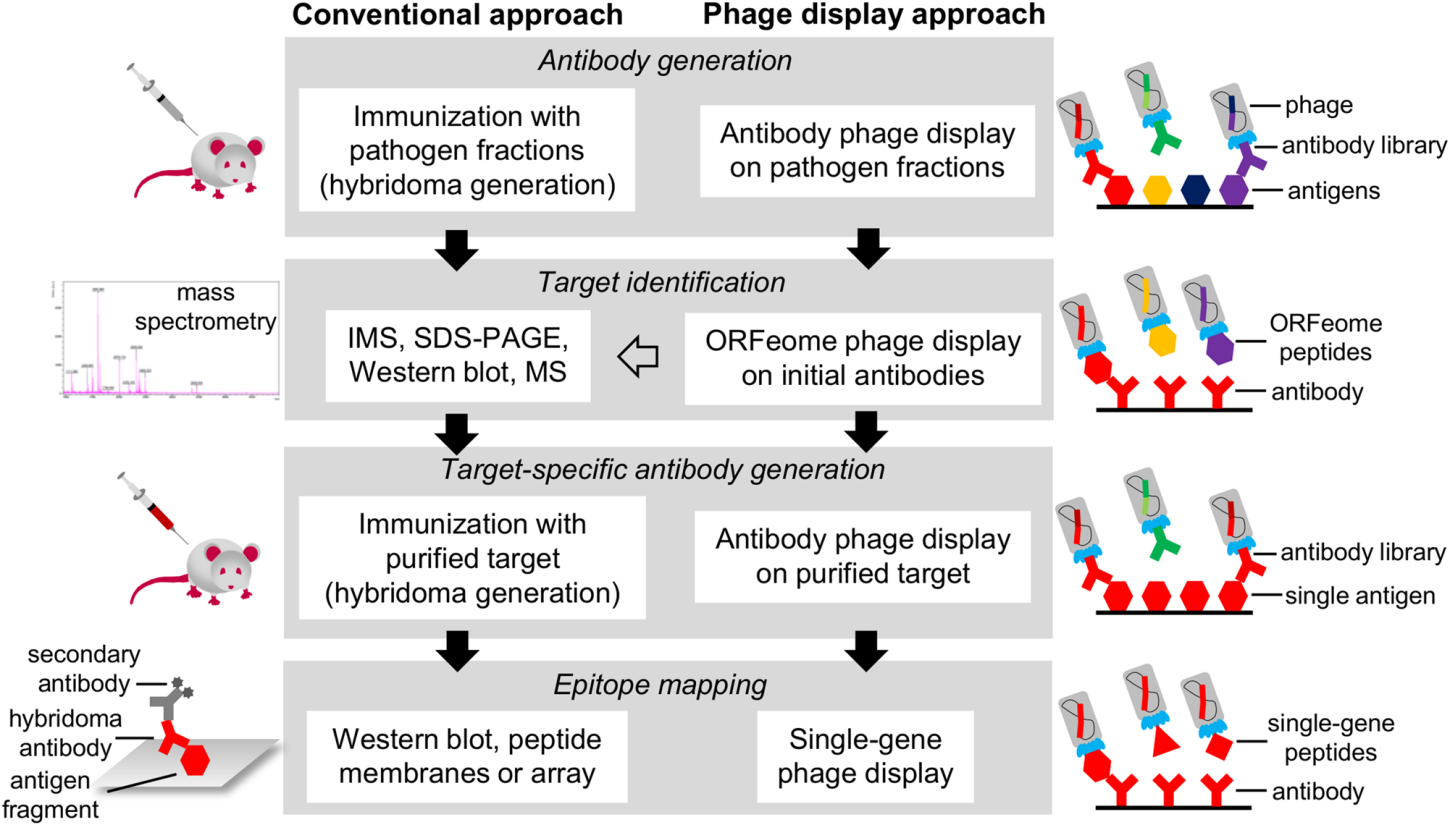
Representation of the workflow of the phage display technologies used in this work compared to conventional techniques. In studies aiming to develop antibodies and find new biomarkers, some standard steps must be followed to characterize either the generated antibodies or the discovered antigen. These steps can be achieved with conventional techniques (left workflow), such as hybridoma technology, IMS, MS, and peptide membranes or arrays, which have been successfully applied. In this study, the conventional techniques could be replaced by one of the corresponding phage display methods, which are free of animal use, according to the desired purpose (right workflow). In addition, IMS-MS was used in parallel to validate the results from ORFeome phage display (gray arrow).

Another application of phage display is for the display of entire ORFeomes and metagenomes. This technique, which employs a library built with antigen fragments from bacterial genomes, metagenomes, or cDNA, was described to allow the discovery of novel oligopeptide markers using sera or other sources of polyclonal antibodies^40,41,57,58^. Even though this ORFeome application could be used in the present study using polyclonal antibodies against *Listeria*, it would provide information about immunogenic proteins that are applicable for indirect detection. However, since the aim was to develop a direct detection, antibody phage display was used in place of ORFeome in order to acquire antibodies that are able to detect targets in intact cells. Nevertheless, ORFeome was employed for target identification, which otherwise is typically achieved by MS-based techniques. In comparison to the IMS-MS approach, ORFeome phage display identified the same target. In this approach, 3 out of the 4 antibodies showed the same target as the IMS-MS (Table S2), suggesting that ORFeome phage display may provide similar information than IMS-MS regarding target identification, allowing a “phage display-only” approach (Fig. 5).

After performing the MSR analysis using single-gene phage display, most of the antibodies showed MSR length ranging from 71 and 87 amino acids, referring to the approximate size of one of the LD of PDC-E2. This indicates that the correct folding of these domains is essential for recognition by the selected antibodies, an observation that was also noticed in a previous study with diphtheria toxin, where the MSR of the C-domain had ≈ 150 amino acids^59^. Single-gene panning also showed that GSM133-A4 actually recognized both LD of the target, and made it possible to determine the MSR of GSM134-C1, which showed no result with ORFeome display. Even though ORFeome also has been reported to allow epitope mapping in the case of a hybridoma-derived monoclonal antibody with considerably high resolution^31^, the results from the present study indicate that single-gene display can significantly improve the resolution of the detected epitope region. This corroborates with the fact that most of the antibodies mapped against diphtheria toxin using a similar procedure had relatively short MSR (14–38 amino acids)^59^.

PDC is an enzyme complex that catalyzes the conversion of pyruvate into acetyl-CoA, which is a step that connects glycolysis to the citric acid cycle (CAC). Due to its crucial role for cellular respiration, this complex is conserved among almost every organism and is composed of three or four different proteins^60^. Although keeping the same function, the structure of the complex and its enzymes vary among the different forms of life. In eukaryotes, four enzymes often compose the complex, of which a PDC-E3 binding protein (PDC-E3BP) is not present in prokaryotic cells. In prokaryotes, however, the complex is mostly composed of three enzymes, which also show substantial structural differences between Gram-negative and Gram-positive bacteria^61^. The basic difference between the PDC structures is the number of PDC-E2 copies that compose the core of the complex. In eukaryotes, there are several models proposed for PDC, which can contain 40–60 copies of PDC-E2, together with 12–20 of PDC-E3BP. In Gram-negatives, the core is composed of ≈ 24 copies of PDC-E2, while in Gram-positives, the core can also have ≈ 24 copies (forming an octahedral symmetry, cube shape) or get closer to that of eukaryotes with ≈ 60 copies (forming an icosahedral symmetry, dodecahedron shape). Considering this structural information, mainly for PDC-E2, and the indirect ELISA results showed in this study, it is possible to observe some correlations. The Gram-negative species used in the present study showed no reaction with most of the tested antibodies (only GSM313-B12 showed cross-reactivity), in accordance with the already known structural difference between Gram-negatives and Gram-positives. Additionally, the known structures of *E. faecalis* (species used in this study) and *Geobacillus stearothermophilus* show an icosahedral symmetry; and since *Enterecoccus* was one of the genera showing most of the cross-reactivity in indirect ELISA, it may be that *Listeria* spp. PDC has the same kind of symmetry.

A quite puzzling result of our study was the detection of PDC on the outside of the *Listeria* cells. The PDC in eukaryotes is known to act in the mitochondrial matrix after the transport of pyruvate from the cytosol. However, the exact location of the complex in the matrix is not fully described and, thus, any proximity to membranes in mitochondria is not clear. Similarly, the PDC in Gram-negatives, as well as in Gram-positives, is mostly found in the cytosol. Nevertheless, some other metabolic proteins, such as 1,6-fructose bisphosphate aldolase (FBA), that were described to be mainly cytoplasmic or attached to the membrane, were also found in the cell wall and, thus, accessible on the cell surface^31,62^. PDC-E2 in *Listeria* may behave similarly to FBA, being a protein that is mainly present in the cytoplasm but makes its way through the membrane reaching the cell wall and surface. In the present study, the panning strategies using cell wall, membrane, and cytoplasm were all able to provide binders against PDC-E2, corroborating the assumption of its location in different cellular parts. Further studies could address whether other metabolic proteins that show up on the bacterial surface provide useful biomarkers for detection, especially considering that *Listeria* surface is remodeled according to the environment^63^.

Although the metabolic function of PDC is well defined, this protein may also have a relation to pathogenicity. In *Mycobacterium tuberculosis*, PDC-E2 was found to induce a strong cellular response in an immunocompetent infected population, also contributing to the bacterial resistance against host reactive nitrogen intermediates^64^. In *Mycobacterium bovis*, PDC-E1β was identified as an immunodominant antigen in infected cattle, allowing the development of an indirect ELISA diagnostic test with better performance than commercial assays^65^. The PDC-E1β of *Mycobacterium pneumoniae* was described to possess fibronectin-binding activity, allowing its classification as a moonlighting protein^66^. In another study, PDC-E1 of *Salmonella enterica* subsp. *enterica* serovar Enteritidis was described as a virulence factor, once mutants with deletions or without the gene showed to be less capable of causing important symptoms of the disease^67^. Characteristics such as slower growth and increased membrane fluidity also occur in PDC-deficient *Staphylococcus aureus*, indicating a structural impact of the enzyme^68^. PDC-E2 has been also proposed as a target for antibiotic treatment^69,70^ and vaccine development^71^ for *M. tuberculosis*. However, up to date, no study has evaluated the impact of PDC from *Listeria* spp. in any of these aspects. Since other metabolic enzymes, such as *Listeria* adhesion protein (LAP), were shown to have a moonlighting role^72^, the function of *Listeria* PDC should be further investigated.

Some *Listeria* species showed reduced reactivity with most of the antibodies against PDC-E2 in indirect ELISA, while some non-*Listeria* species had elevated reactivity (Figure S4). *Listeria grayi*, *L. aquatica*, and *L. cornellensis* showed the lowest reaction levels among the *Listeria* species, with signal-to-noise ratio barely above 1. Although many factors not investigated in this study may contribute to the difference in the reactivity, some possibilities and observations may be worth mentioning. *Listeria grayi* is genetically classified in an independent phylogenetic subgroup (called *Murraya*), indicating special characteristics that may affect protein expression profile and composition of the cell surface^73^. In accordance with this, when analyzing the phylogeny based on sequences of PDC-E2, *L. grayi* also appears to be isolated from other species of the genus, showing the highest genetic distance (Figure S5). *Listeria aquatica* is part of another subgroup (called *Mesolisteria*) together with *L. fleischmannii* and *L. floridensis*. Nevertheless, it shares some characteristics with *L. grayi*, as both species are the only two of the genus known to produce acetoin out of glucose metabolism^74^. Additionally, both species show similar genetic changes and the highest genetic distance when compared to the other species using 325 single-copy genes for analysis^73^. Similar behavior is noticed when using PDC-E2 sequences, where both *L. grayi* and *L. aquatica* show the highest genetic distance (Figure S5). As to *L. cornellensis*, it is grouped in a different subgroup (called *Paenilisteria*). It is worth highlighting that the optimal growth of this species in the present study was achieved at 30 °C, instead of 37 °C for most of the other species. Curiously, all the species grown under 30 °C in this study are part of this subgroup and show up in the same phylogenetic cluster^73^. In addition, *L. cornellensis* is the only species that presents a low lactose acidification^74^. As to the three newest *Listeria* species, which could not yet be included in this study, it is expected that the two best antibodies against PDC-E2 could very well also recognize them since the identity of their PDC-E2 compared to that of the target with that of *L. monocytogenes* is 82.5% or higher (see Table S1).

When analyzing the reaction pattern of the non-*Listeria* strains, it is clear that *Bacillus* and *Enterococcus* species show elevated reactivity with some of the antibodies. Accordingly, many studies describe *Bacillus* as closely related to *Listeria*, strongly indicating phenotypic similarities^75,76^. Although phylogenetic comparisons between *Listeria* and *Enterococcus* are not often made, these organisms may be related enough to share a phenotype that could explain the high reactivity with some of the antibodies^77^. Additionally, PDC-E2 of *Bacillus* and *Enterococcus* show considerable identity to that of *L. monocytogenes* (> 50%), which may explain the cross-reactivity. The fact that only Gram-positive species showed such reactions indicates a higher tendency of PDC-E2 to be exposed on the cell surface, maybe as a consequence of the similar structure of PDC^61^. In summary, the recognition of PDC-E2 by the antibodies shown in this study may depend on multiple factors, such as the cellular location of the target (phenotype), identity to *Listeria* PDC-E2, and the overall structure of the complex.

It is interesting to note that the recognition of both LD of PDC-E2 was not beneficial for the diagnostic performance. It is expected that the recognition of both LD in the same molecule would result in a higher signal, which may lead to higher sensitivity. Nonetheless, although GSM313-F7 shows stronger reactions than most of the antibodies, the increase in reactivity seems not to be a trend of the antibodies that recognize both LD and does not contribute to the sensitivity (Figure S6). In addition, the recognition of both LD may be associated with reduced specificity, since 2 out of the 4 scFv-Fc with this pattern of recognition show elevated reactions with non-*Listeria* species and AUC < 0.8 (0.7895 for GSM313-F7, and 0.6632 for GSM313-G10), placing them in the bottom five positions in respect of diagnostic performance (see Table 2; the other two antibodies were GSM313-D10 and GSM133-A4, which showed AUC = 0.9789 and 0.8947, respectively).

The two scFv-Fc with best diagnostic performances (GSM313-E9 and GSM313-H8) showed an interesting pattern, as they recognize the 1st LD and a synthetic LD formed by the fusion of the two LD of PDC-E2, which was probably a result of the random fragmentation and ligation during the library construction. Another interesting antibody is GSM130-H1 since it was the best performing antibody out of the initially generated against protein fractions. It is worth mentioning that this antibody was selected out of the panning strategy using *Listeria* cell wall proteins, which indicates that its better performance could be connected to the recognition of PDC-E2 in a specific conformation it assumes in this subcellular fraction. The case of GSM133-E2 is also worth mentioning, since it is specific for PDC-E2, but presents multiple binding sites, possibly exemplifying a case of multispecificity. Multispecific antibodies are known to recognize more than one epitope with high specificity^78–80^. The detected peptides from outside the LD regions only slightly higher hydrophobic composition (45–55% of the amino acids, compared to ≈ 40% in the LD regions), but not excessively elevated considering that highly hydrophobic peptides often responsible for unspecific reactions typically contain > 50% of hydrophobic amino acids and < 25% acidic composition^81^. This way, it is probable that GSM133-E2 is not intrinsically unspecific, but shows a multispecificity that lowers its diagnostic performance. If this were the case, this antibody could bind different epitopes of the same target instead of similar epitopes in different targets.

The combination of in vitro technologies described here (antibody display, ORFeome display, and single-gene display, see Fig. 5) allowed to identify a novel biomarker (PDC-E2), generate monoclonal antibodies that are potentially useful for detection of *Listeria* spp. in food (GSM313-E9 and GSM313-H8), and characterize the underlying antibody-antigen interactions. This way, the list of useful targets and biomolecules for the detection of this genus is increased. Further studies are necessary to evaluate these molecules in commercially applicable tests.

## Methods

### Bacteria cultivation and *Listeria* protein fractionation

The proteins from the cell wall, membrane, and cytoplasm from *Listeria* spp. cells were prepared as described before^82^. ELISA was used to determine the presence of proteins InlA (present in the cell wall) and FBA (mainly present in cytoplasm and membrane). For this, the cell wall, cytoplasm, and membrane fractions were diluted in phosphate-buffered saline (PBS) 1:100, 1:100, and 1:400, respectively, and were coated on ELISA Costar plates (Corning). Plates were blocked with 2% (w/v) milk powder diluted in PBS supplemented with 0.05% (v/v) Tween-20 (2% MPBS-T), and incubated with monoclonal antibodies 2D12 (mouse IgG anti-InlA, 1 µg/mL)^22^ and 3F8 (mouse IgM anti-FBA, 1 µg/mL)^31^. Finally, the plates were incubated either with anti-mouse IgG Fc specific (Sigma) for 2D12, or anti-mouse IgA, G, M (Antibodies Online) for 3F8, both HRP conjugated. The reaction was developed with TMB solution (TMB-A: 50 mM citric acid, 30 mM potassium citrate, pH 4.1; TMB-B: 90% (v/v) ethanol, 10% (v/v) acetone; 10 mM tetramethylbenzidine; 1 mL 30% H_2_O_2_; mix 19 parts of TMB-A with 1 part of TMB-B), stopped with 1 N H_2_SO_4_, and plates were analyzed at 450 nm, using 620 nm as reference.

### Antibody panning on *Listeria* protein fractions or rPDC-E2, and monoclonal scFv screening

The *Listeria* protein fractions were used in different panning strategies. In total, four approaches were performed using the human naïve antibody phage display libraries HAL9 and HAL10^83^ separately. All these strategies started with pre-incubations of the libraries packaged with Hyperphage (Progen) in three ELISA wells with a combination of panning block solution (1% milk powder, 1% BSA diluted in PBS-T), *L. innocua* DSM 20649 cell wall, or heat-inactivated *Bacillus subtilis* 168 NCIB 10106. Heat-inactivated *B. subtilis* cells were prepared by growing in BHI overnight at 37 °C, 250 RPM. Cells were harvested (2,600×*g*, 7 min, 4 °C), suspended in carbonate-bicarbonate buffer (75 mM Na_2_CO_3_, 75 mM NaHCO_3_, pH 9.7) until OD_600_ = 1.0 (≈ 10^9^ cells/mL), heated for 10 min at 90 °C, and stored at − 20 °C until use. The following panning strategies were carried out: (1) *L. monocytogenes* ATCC 7644 cell wall fraction, with pre-incubation on *B. subtilis* (1 ×) and PanningBlock (2 ×); (2) *L. monocytogenes* ATCC 7644 cytoplasm fraction, with the same pre-incubation; (3) *L. monocytogenes* ATCC 7644 membrane fraction, with the same pre-incubation; (4) same as strategy “1”, but with pre-incubation on *L. innocua* DSM 20649 cell wall.

For screening, cell wall, membrane, and cytoplasm fractions were coated as described above. The monoclonal scFv produced were tested against the protein fractions, *L. monocytogenes* ATCC 7644, *L. innocua* DSM 20649, or *B. subtilis* 168 NCIB 10106 for negative control. The remaining procedure was conducted as previously published^84^.

A similar procedure was made for the panning against recombinant PDC-E2. This time, however, libraries HAL9 and HAL10 were mixed in a single panning well coated with 1 µg of protein diluted in PBS. For screening, a plate with 200 ng/well of the recombinant protein diluted in PBS was used in place of the protein fractions.

### Cloning and production of scFv-Fc and IgG

Selected scFv against protein fractions from the previous step had their genes subcloned into pCSE2.6 vectors with mouse IgG2a Fc for scFv-Fc production. In the case of GSM133-E2, it was also produced as human IgG1 using the vectors pCSEH and pCSL^85^. Cloning and production in HEK293 cells were performed as previously described^86^. After purification, the binding of every scFv-Fc antibody on *Listeria* spp. cells was checked by ELISA, similarly to the scFv screening. This time, the antibodies were diluted using √10-fold series.

### Immunoblot with protein fractions, and target characterization by mass spectrometry

The protein fractions from the cell wall and cytoplasm of *L. innocua* DSM 20649 were separated by 12% SDS-PAGE and subsequently transferred to a methanol-activated PVDF 0.45 µm membrane (Roth). The membrane was blocked with 2% MPBS-T overnight, and further incubated with 1 µg/mL of each of the scFv-Fc for 1 h at RT. Goat anti-mouse IgG Fc-specific HRP-conjugated (1:40,000; Sigma) was used as a secondary antibody, and DAB solution (6 mg 3.3-diaminobenzidine tetrahydrochloride; 10 μL 30% H_2_O_2_; 9 mL PBS; 1 mL NiSO_4_ 250 mM) solution was finally added for 15 min.

Immunomagnetic separation (IMS) was performed with 100 µL of SureBeads Protein A magnetic beads (Bio-Rad), which were coated with 10 µg of each antibody for 10 min. A cytoplasmic preparation of *L. innocua* DSM 20649 was diluted 1:2 in PBS in a total of 200 µL and incubated with the beads for 1 h. Subsequently, the elution was made with glycine buffer 20 mM, pH 2.0 for 5 min. Then, 10% (v/v) Tris-NaCl 1 M, pH 7.4 was added. The elution samples were analyzed in SDS-PAGE, from which the proteins referring to the target were excised for mass spectrometry (MS) analysis. The corresponding immunoblot was performed the same way as described above, using as primary antibody the same scFv-Fc used for IMS.

The bands taken from SDS-PAGE gel were processed as previously described^87^. Purified peptides were then eluted directly and co-crystallized with α-cyano-4-hydroxycinnamic acid onto anchor chip targets (Bruker) for matrix-assisted laser desorption ionization (MALDI). MS analysis was performed manually with Ultraflex time-of-flight (TOF) and TOF mass spectrometer (Bruker). The peptides were identified in a peptide mass fingerprinting approach MS data processing was performed with Bruker's Biotools and in house Mascot server (Matrix Science v. 2.4.1). Database searches were restricted to the genus *Listeria* of the National Center for Biotechnology Information database (NCBI, version 20170811) and methionine oxidation was considered as a variable, as well as fixed as iodoacetamide modifications.

### ORFeome display

A phage library built from random genome fragments of *L. monocytogenes* ATCC 7644 was used to perform panning on obtained antibodies (GSM130-H1, GSM133-A4, GSM133-E2, and GSM134-C1) in order to identify the antibodies’ targets. The used library was described in a previous study^31^. Briefly, 1 µg of the antibodies was coated on an ELISA plate. For pre-clearance of the library, 2 × 10^11^ CFU was incubated 20 min at RT on each of the other three different wells: (1) PanningBlock solution; (2) a non-related scFv-Fc; and (3) a non-related scFv-Fc plus 10 µg of the scFv-Fc in solution. The remaining steps were done as previously described, except that only one panning round was conducted. After identifying reactive clones by ELISA, they were sequenced and analyzed with BLASTn.

In order to confirm and validate the results from screening ELISA, positive hits were produced as monoclonal phage on a larger scale using previously described procedure^43^. The final suspension containing produced monoclonal phage was titrated in indirect ELISA to confirm the binding to each antibody. For this, phage were coated onto ELISA wells, diluting them 12 times √10-fold starting with 10^9^ CFU/well. The antibodies against the respective monoclonal phage were adjusted to 2 µg/mL and incubated for 1 h with goat anti-mouse IgG Fc-specific HRP-conjugated antibody (1:40,000; Sigma).

### Microscopy of *Listeria* cells

The strain *L. monocytogenes* ser. 4b DSM 15675 was grown in BHI (16 h; 37 °C; 110 RPM). Then, 2 mL were centrifuged (2,500×*g*; 2 min), washed 2 times with PBS, and incubated with three different antibodies: (1) anti-internalin A scFv-Fc as a positive control; (2) a non-related scFv-Fc as a negative control; or (3) GSM133-A4 scFv-Fc (anti-PDC-E2, as experiment molecule). These antibodies were diluted in 2% MPBS-T (5 µg/mL final concentration) and incubated 1 h at RT. Cells were washed three times with PBS-T and incubated 1 h at RT with anti-mouse IgG Alexa 647 secondary antibody (Thermo Scientific), diluted 1:500 in 2% MPBS-T. Cells were washed the same way and suspended in a corresponding volume DAPCO (1,4-diazabicyclo[2.2.2]octane) containing mounting medium. Sample preparation was performed as described before^88^. Imaging was performed with a Nikon Eclipse Ti inverse microscope with Cy5 (650/13–705/72) filters. Fluorescence z-stacks and phase-contrast images were taken using a Nikon N Plan Apochromat l λ × 100/1.45 oil objective and the ORCA FLASH 4.0 HAMMATSU camera. Images were processed using the NIS-elements imaging software V4.3 (Nikon) together with the 3D Landweber Deconvolution algorithm.

### Cloning, expression in *E. coli*, and purification of the recombinant target PDC-E2

The gene (code: AL596167.1:98629-100260) and protein (code: WP_010990728.1) sequences of PDC-E2 were obtained from GenBank and used to design primers for subcloning. Primers containing the restriction sites *Nde*I and *Not*I were ordered as follows: forward 5′-AATTCCATATGGCATATTCATTTAAATTACCGGATATCG-3′, and reverse 5′-ATTGCGGCCGCCACCTCCATTAGTAATAATTCTG-3′. The PCR was conducted with Phusion High-Fidelity DNA Polymerase (Thermo Scientific), according to the manufacturer’s instructions, using the genomic DNA of *L. monocytogenes* ATCC 7644. The amplified gene was subcloned into pET21a(+) (Novagen).

For the expression, *E. coli* BLR(DE3) containing pET21a(+)/PDC-E2 was grown in 10 mL LB-A (Luria–Bertani containing 100 µg/mL ampicillin) for 16 h at 37 °C, 250 RPM. Then, 200 mL of LB-A was inoculated until OD_600_ = 0.1 and grown under the same conditions until OD_600_ = 0.6–0.8, when it was induced with IPTG to a final concentration of 125 µM and incubated for 4 h. The cells were harvested by centrifugation (10,000×*g*; 10 min; 4 °C), suspended in 20 mL binding buffer (5 mM imidazole, 0.5 M NaCl, 20 mM Tris–HCl, pH 7.9), and sonicated (3 × 2 min of pulsed cycles). The suspension was centrifuged (16,000×*g*; 10 min; 4 °C) and the supernatant transferred to a new tube. Electrophoresis and immunoblot with anti-6xHis antibody (Dianova) were performed to confirm the expression. Finally, the Ni^2+^-affinity purification was made in a gravity-flow column (GE Healthcare) using washing (60 mM imidazole, 0.5 M NaCl, 20 mM Tris–HCl, pH 7.9) and elution buffers (0.5 M imidazole, 0.5 M NaCl, 20 mM Tris–HCl, pH 7.9). The protein was dialyzed against elution buffer diluted with PBS to gradually decrease the concentration of NaCl as previously published^89^.

### Indirect ELISA for *Listeria* spp. detection

The employed strains (Tables 4 and 5) were obtained from the Leibniz Institute DSMZ-German Collection of Microorganisms and Cell Cultures. Each bacterium was recovered from lyophilized stocks with BHI, grown in 10 mL BHI, and streaked onto BHI-agar plates. The exception was *L. paracasei*, which was always grown in MRS or MRS-agar. Bacteria were grown according to their handling description at 30 or 37 °C, shaking at 80 or 110 RPM, respectively. When needed, microaerobic conditions were created by using anaerobic candle jars.

**Table 4:** List of the *Listeria* species used for indirect ELISA with antibodies against PDC-E2.

Species	Serovar	DSM no.	Temperature (°C)	Condition
*L. monocytogenes*	4b	15675	37	Aerobic
	1/2a	102976	37	Aerobic
	1/2b	19094	37	Aerobic
*L. innocua*	6a	20649	37	Aerobic
*L. marthii*	NI	23813	37	Aerobic
*L. welshimeri*	1/2b	20650	37	Aerobic
*L. ivanovii*	5	20750	37	Aerobic
*L. seeligeri*	1/2b	20751	37	Aerobic
*L. floridensis*	NI	26687	37	Aerobic
*L. fleischmannii *subsp*. fleischmannii*	NI	24998	37	Aerobic
*L. aquatica*	NI	26686	37	Aerobic
*L. grayi*	NI	20601	37	Aerobic
*L. cornellensis*	NI	26689	30	Aerobic
*L. rocourtiae*	NI	22097	30	Aerobic
*L. booriae*	NI	28860	37	Aerobic
*L. riparia*	NI	26685	37	Aerobic
*L. weihenstephanensis*	NI	24698	30	Aerobic
*L. grandensis*	NI	26688	30	Aerobic
*L. newyorkensis*	NI	28861	37	Aerobic

*NI* not informed.

**Table 5:** List of the non-*Listeria* species used for indirect ELISA with antibodies against PDC-E2.

Species	Serovar	DSM no.	Temperature (°C)	Condition
*Salmonella enterica*	Typhimurium	17058	37	Aerobic
*Escherichia coli*	O157:H7	17076	37	Aerobic
*Pseudomonas aeruginosa*	NI	50071	37	Aerobic
*Klebsiella pneumoniae*	3	30104	37	Aerobic
*K. aerogenes*	NI	30053	30	Aerobic
*Enterobacter cloacae*	NI	30054	30	Aerobic
*Staphylococcus aureus*	3	20231	37	Aerobic
*Jonesia denitrificans*	NI	20603	37	Aerobic
*Bacillus subtilis*	NI	10	30	Aerobic
*B. thuringiensis*	NI	2046	30	Aerobic
*B. cereus*	NI	31	30	Aerobic
*Enterococcus faecium*	D, 11	20477	37	Microaerobic
*E. faecalis*	D	20478	37	Microaerobic
*E. lactis*	NI	23655	37	Microaerobic
*Lactococcus lactis*	N	20481	30	Microaerobic
*Lactobacillus paracasei*	NI	5622	30	Microaerobic

*NI* not informed.

For the ELISA, a single colony of each strain was taken from the plates and grown in 10 mL of the corresponding medium under the standard conditions (Tables 4 and 5). Then, all cells were collected by centrifugation, washed twice with PBS, and suspended in carbonate-bicarbonate buffer to an OD_600_ = 1.0. ELISA plates were prepared by coating with 100 µL/well of the cell suspension at 4 °C overnight. Plates were blocked with 2% MPBS-T for 1 h at RT, and further incubated 1 h at RT with the different antibodies diluted in 2% MPBS-T in three different concentrations: (a) the estimated EC_50_ (“EC_50_”); (b) a √10 dilution above the EC_50_ (“EC_50_+”); and (c) a √10 dilution below the EC_50_ (“EC_50_−”). As a secondary antibody, goat anti-mouse IgG Fc-specific HRP-conjugated (1:30,000; Jackson ImmunoResearch Laboratories) was used. Wells with a non-related scFv-Fc, diluted √10-fold starting with 10 µL/mL, and wells containing only the secondary antibody served as controls.

The signal-to-noise ratio was calculated from every reading divided by the average of three wells from the secondary antibody control of each plate. Every combination of the antibody concentration and bacterial strain was performed in a single well that was repeated twice in independent plates, although only one of the plates was selected to perform the statistical analysis. The resulting values were used in a Receiver Operating Characteristic (ROC) analysis using GraphPad software (Prism, v 5.01), in which the sensitivity and specificity, as well as the confidence intervals (CI), were calculated. To facilitate the description results, the area under curve (AUC) value was also used for representing this data.

### Single-gene phage display of PDC-E2

A single-gene library was built with the gene coding for PDC-E2, as described previously^43,44^. The panning was performed similarly to the ORFeome panning for target discovery, where 1 µg of each antibody was coated. Per panning well, 10^9^ CFU were used and only the first panning round was conducted. The screening was done as described above and reactive clones were sent for sequencing. The resulting sequences were aligned with ClustalOmega software to define the minimal sequence of recognition (MSR), defined as the shortest sequence present on every selected clone.

### Sequence analysis of PDC-E2

The protein sequences of the target PDC-E2 detected with MS and ORFeome phage display were used in BLASTp to find the homologs from each of the species used in the indirect ELISA. Identity and similarity were calculated with the online pairwise alignment tool EMBOSS Needle^90^ (Table S2) using the sequence of PDC-E2 (GenBank code: WP_107899613.1) as the query. This same sequence was analyzed with SMART online software^91^, which allowed defining its different regions.

## Supplementary information

Supplementary Information.

## Data Availability

The authors agree that the data presented here can be publicly available.
