# Supplementary material for: Pyruvate dehydrogenase complex—enzyme 2, a new target for Listeria spp. detection identified using combined phage display technologies
Source: Sci Rep. 2020 Sep 17;10:15267. doi: 10.1038/s41598-020-72159-4 (PMC7498459; doi:10.1038/s41598-020-72159-4)
Supplement: Supplementary file 1 — Supplementary Information. [file 41598_2020_72159_MOESM1_ESM.pdf]

Supplementary Information

Pyruvate dehydrogenase complex - enzyme 2, a new target for *Listeria* spp. detection identified using combined phage display technologies

Gustavo Marçal Schmidt Garcia Moreira[1], Sarah Mara Stella Köllner[1], Saskia Helmsing[1], Lothar Jänsch[2], Anja Meier[2], Sabine Gronow[3], Christian Boedeker[3], Stefan Dübel[1], Marcelo Mendonça[4], Ângela Nunes Moreira[5], Fabricio Rochedo Conceição[5], Michael Hust[1*]

[1]Technische Universität Braunschweig, Institut für Biochemie, Biotechnologie und Bioinformatik, Abteilung Biotechnologie, Braunschweig, Lower Saxony, Germany
[2]Cellular Proteomics, Helmholtz Centre for Infection Research, Braunschweig, Lower Saxony, Germany
[3]Leibniz Institute DSMZ-German Collection of Microorganisms and Cell Cultures, Braunschweig, Lower Saxony, Germany
[4]Universidade Federal Rural de Pernambuco, Unidade Acadêmica de Garanhuns, Garanhuns, PE, Brazil
[5]Laboratório de Imunologia Aplicada, Núcleo de Biotecnologia, Centro de Desenvolvimento Tecnológico, Universidade Federal de Pelotas, Pelotas, RS, Brazil

* Correspondence: Michael Hust, m.hust@tu-braunschweig.de

1 Tables

Table S1. Summary of the hits found by mass spectrometry or ORFeome display.

Technique	GenBank code	Sequence coverage (%) / Score	Protein information [b]
Mass spectrometry [a]	WP_010990728.1	43.0 / 151	dihydrolipoamide acetyltransferase, *Listeria innocua* (target)
	WP_072572643.1	49.0 / 155	pyruvate dehydrogenase (acetyl-transferring) E1 component subunit alpha, *Listeria monocytogenes* (unknown 2)
	WP_038409535.1	56.0 / 191	alpha-ketoacid dehydrogenase subunit beta, *Listeria monocytogenes* (unknown 3)
ORFeome phage display	WP_107899613.1 [c]	NA	dihydrolipoamide acetyltransferase, *Listeria monocytogenes* (GSM130-H1, GSM133-A4, GSM133-E2)

[a] The sequences were identified by MASCOT software.

[b] For mass spectrometry, the protein information also contains the protein identification used in Figure 3 of the main article in parenthesis. For ORFeome phage display, the antibodies used for target identification, also shown in Figure 3, are in parenthesis.

[c] This code refers to the cloned sequence used in further steps, which was present in the BLASTx results of the output sequences for the mentioned antibodies.

NA, not applicable.

Table S2. GenBank codes, identity and similarity of the PDC-E2 sequences from the organisms used in indirect ELISA.

Organism	GenBank code	Identity (%)	Similarity (%)	Gaps (%)
Listeria monocytogenes[a,*]	WP_107899613.1	100.0	100.0	None
L. innocua[b]	WP_010990728.1	99.3	99.4	None
L. marthii[c]	EFR88158.1	98.2	98.9	None
L. welshimeri	WP_011701854.1	98.2	98.7	None
L. ivanovii	WP_014092472.1	97.1	98.2	None
L. seeligeri	WP_012985326.1	96.1	97.6	None
L. floridensis	WP_036096782.1	83.2	89.6	1.8
L. fleischmannii subsp. fleischmannii	WP_007472784.1	83.0	89.4	2.7
L. aquatica	WP_036071619.1	82.7	89.6	2.6
L. grayi	WP_036106310.1	82.4	88.5	2.2
L. cornellensis	WP_036077440.1	82.0	89.5	2.5
L. rocourtiae	WP_036070034.1	82.0	89.3	2.2
L. booriae	WP_036086614.1	81.9	89.5	3.2
L. riparia	WP_036098974.1	81.9	89.5	3.2
L. weihenstephanensis	WP_036063351.1	81.9	89.3	2.9
L. grandensis	WP_036067464.1	81.9	89.1	2.9
L. newyorkensis	WP_059140683.1	81.9	88.9	2.9
L. goaenensis[e]	WP_099223139.1	83.0	91.0	1.5
L. costaricensis[e]	WP_088815148.1	83.1	89.9	1.7
L. thailandensis[e]	WP_122865401.1	82.5	89.6	3.1
Salmonella enterica	WP_073877127.1	36.3	54.7	10.6
Escherichia coli	WP_057699170.1	35.4	53.3	11.1
Pseudomonas aeruginosa	WP_042930711.1	35.0	53.5	6.9
Klebsiella pneumoniae	KMH79087.1	34.6	53.4	10.6
K. aerogenes	WP_058655303.1	34.9	52.9	10.6
Enterobacter cloacae	WP_023480469.1	30.7	47.2	21.4

Staphylococcus aureus[d]	WP_061644423.1	51.8	60.8	24.8
Enterococcus faecalis	WP_010827359.1	62.9	73.9	5.2
E. faecium	WP_104674310.1	60.7	73.2	5.2
E. lactis	NF	NF	NF	NF
Lactobacillus paracasei	WP_003598571.1	55.7	70.7	6.6
Bacillus cereus	WP_078181369.1	52.3	62.6	23.4
B. thuringiensis	WP_000863429.1	51.7	63.3	23.4
B. subtilis	WP_060398546.1	50.4	61.9	24.6
Lactococcus lactis	WP_011675116.1	44.4	62.1	4.7
Jonesia denitrificans	WP_015772709.1	26.3	39.7	30.9

[a] Sequence found via ORFeome display followed by BLASTp.

[b] Sequence found with MS.

[c] Sequence is “partial”.

[d] *S. aureus* was not included in the indirect ELISA experiments due to protein A-related background on every well.

[e] This species was not included in this study.

NF, not found with BLASTp.

* *L. monocytogenes* sequence was used as a reference in the alignment.

2 Material and Methods

Western blot and ELISA of scFv-Fc generated against recombinant PDC-E2

The Western blots were done the same way as when using the *Listeria* protein fractions, but this time 1 µg of the purified recombinant protein was run into each lane of a 12 % SDS-PAGE gel (S1 Figure). As a positive control antibody, a mouse anti-6xHis monoclonal (1:500 in 2 % MPBS-T, Dianova) was used. The ELISA was performed by coating 200 ng/well of the recombinant target diluted in PBS overnight at 4 °C. In parallel, another recombinant protein was coated the same way for negative control. After blocking the wells with 2 % MPBS-T, each of the produced antibodies was diluted √10-fold 8 times starting with 10 µg/mL and incubated 1 h at RT. In both assays, goat anti-mouse Fc specific HRP-conjugated (1:40,000; Sigma) was used as a secondary antibody, while SuperSignal West Pico (Thermo Scientific) and TMB solution substrates were used for Western blot and ELISA, respectively.

3 Figures

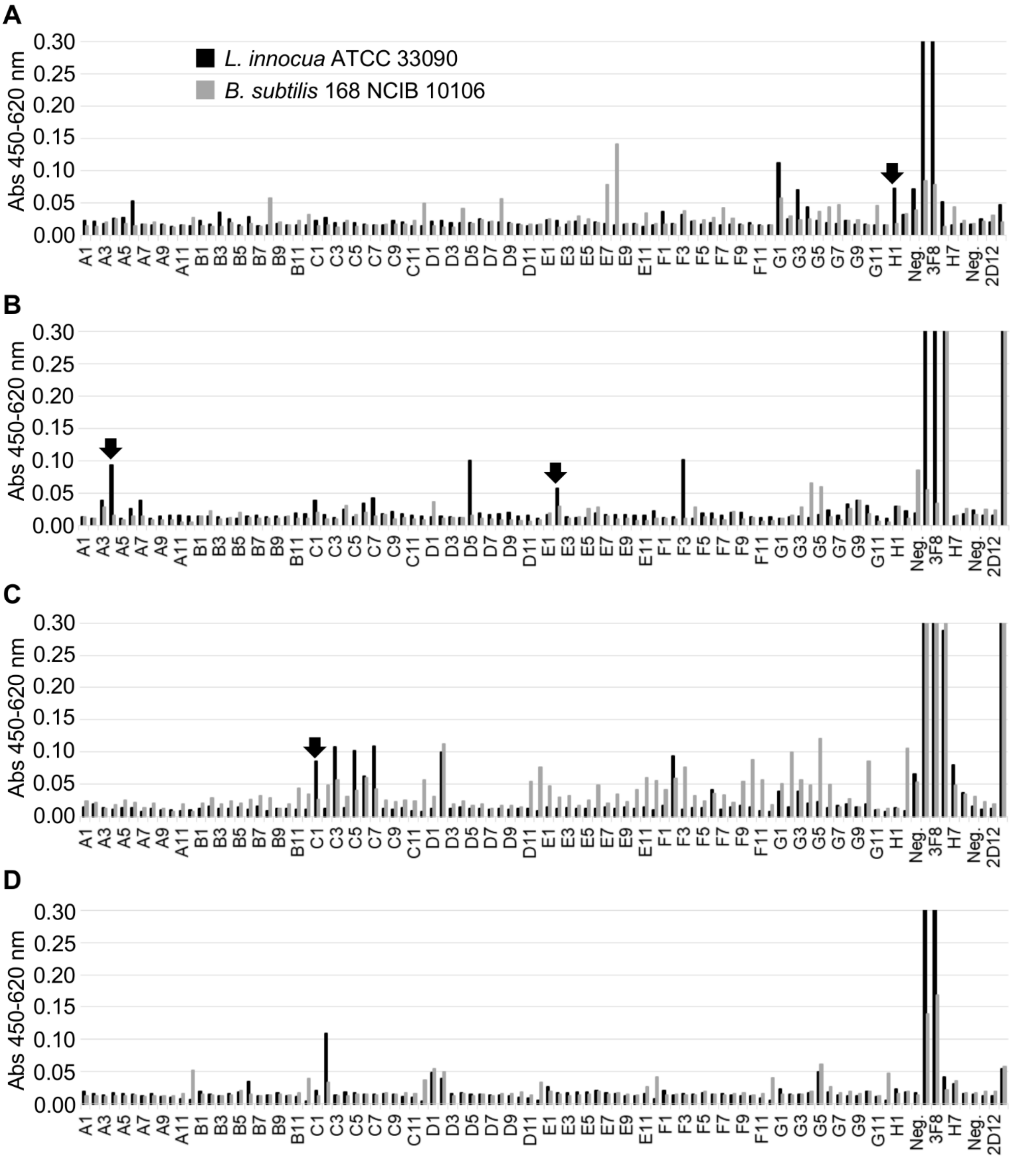

Figure S1. Screening results after performing the panning strategies with *Listeria* protein fractions. The panning strategies employed protein fractions from the cell wall, membrane, and cytoplasm in order to increase chances to acquire antibodies against exposed proteins, mainly from cell wall fractions. From each of the four panning strategies, 92 clones were tested against the respective protein fraction and different bacteria (*L. monocytogenes*, *L. innocua*, and *B. subtilis*). The figure shows the most evident data for the detection of *Listeria* spp., which consists of using *L. innocua* (black bars) as the target, and *B. subtilis* (gray bars) as the negative control. Even though some of the presented graphics show more reactive antibodies than those four initially selected (black arrows), they were discarded either because they had the same sequence, or due to unspecific reaction with *B. subtilis* in titration ELISA. (**A**) Screening results from panning using *Listeria* cell wall proteins (strategy 1), which led to the identification of GSM130-H1. (**B**) Screening results from panning using *Listeria* cytoplasm proteins (strategy 2), which led to the identification

of GSM133-A4 and GSM133-E2. (**C**) Screening results from panning using *Listeria* membrane proteins (strategy 3), which led to the identification of GSM134-C1. (**D**) Screening results from panning using *Listeria* membrane proteins (strategy 4). This was the only strategy leading to no relevant antibodies for detection.

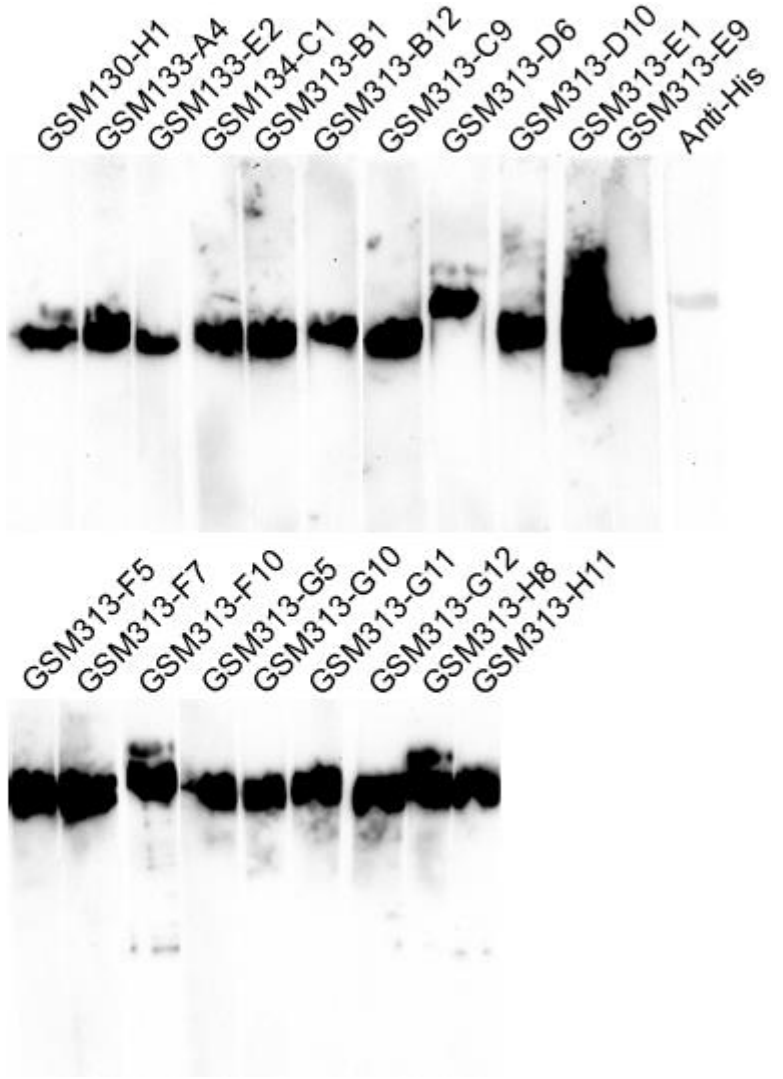

Figure S2. Immunoblot of the scFv-Fc antibodies generated against the purified recombinant PDC-E2. The 20 newly generated scFv-Fc were diluted to 1 µg/mL and tested against 1 µg of the recombinant PDC-E2. Of note, the immunoblot shown is an assembly of different stripes of a membrane, which may lead to the not exact position of the band.

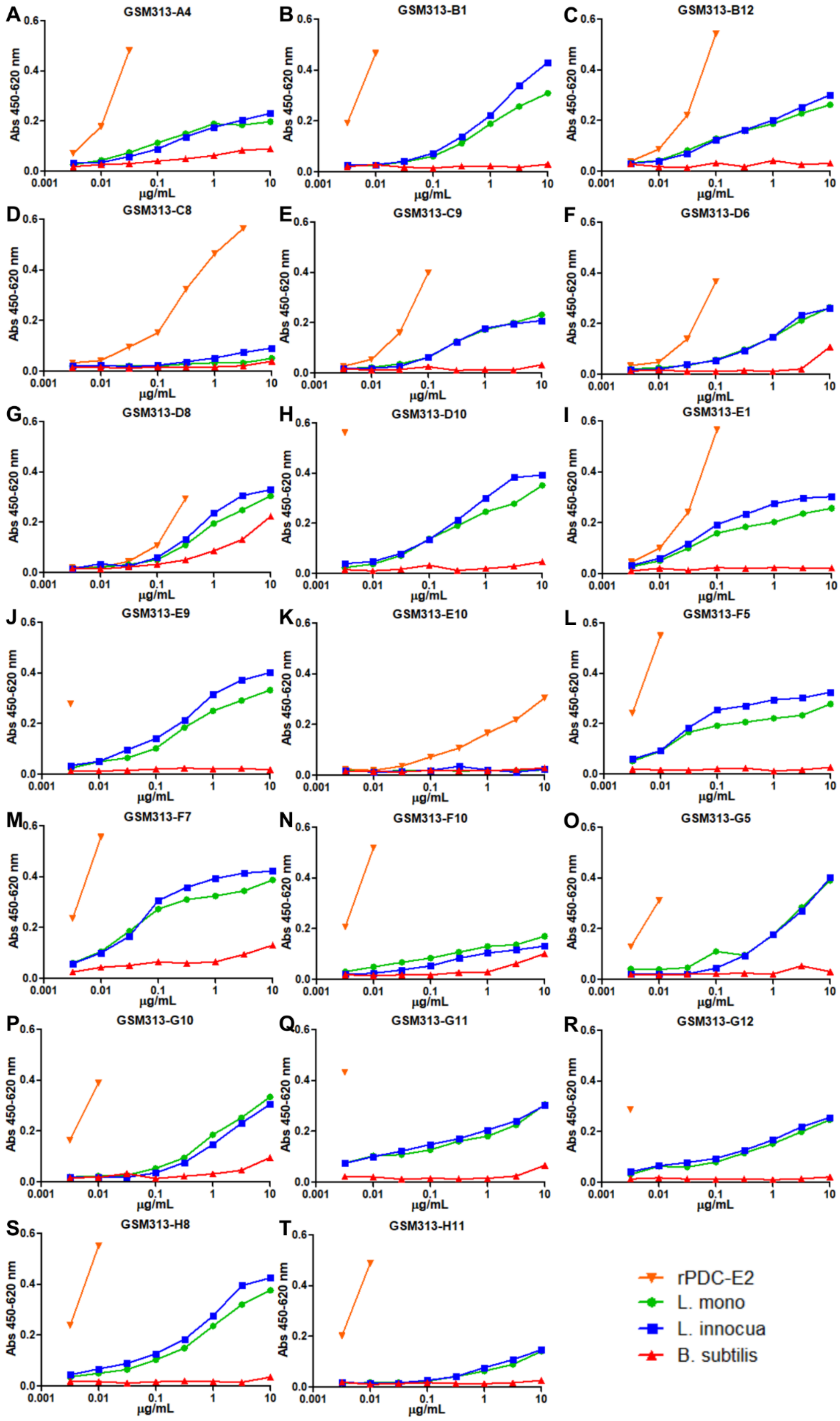

Figure S3. ELISA of the scFv-Fc antibodies generated against the purified recombinant PDC-E2. The 20 newly generated scFv-Fc (**A-T**) were diluted √10-fold from 10 µg/mL until 3.2 ng/mL. Then, they were tested against the recombinant PDC-E2 (rPDC-E2, orange) and three strains coated alive onto ELISA plates: *L. monocytogenes* ATCC 7644 (L. mono, green), *L. innocua* DSM 20649 (L. innocua, blue), and *B. subtilis*

168 NCIB 10106 (B. subtilis, red). Of note, some points of the titration with rPDC-E2 are not represented in order to have a better visualization of the reactions with the bacteria.

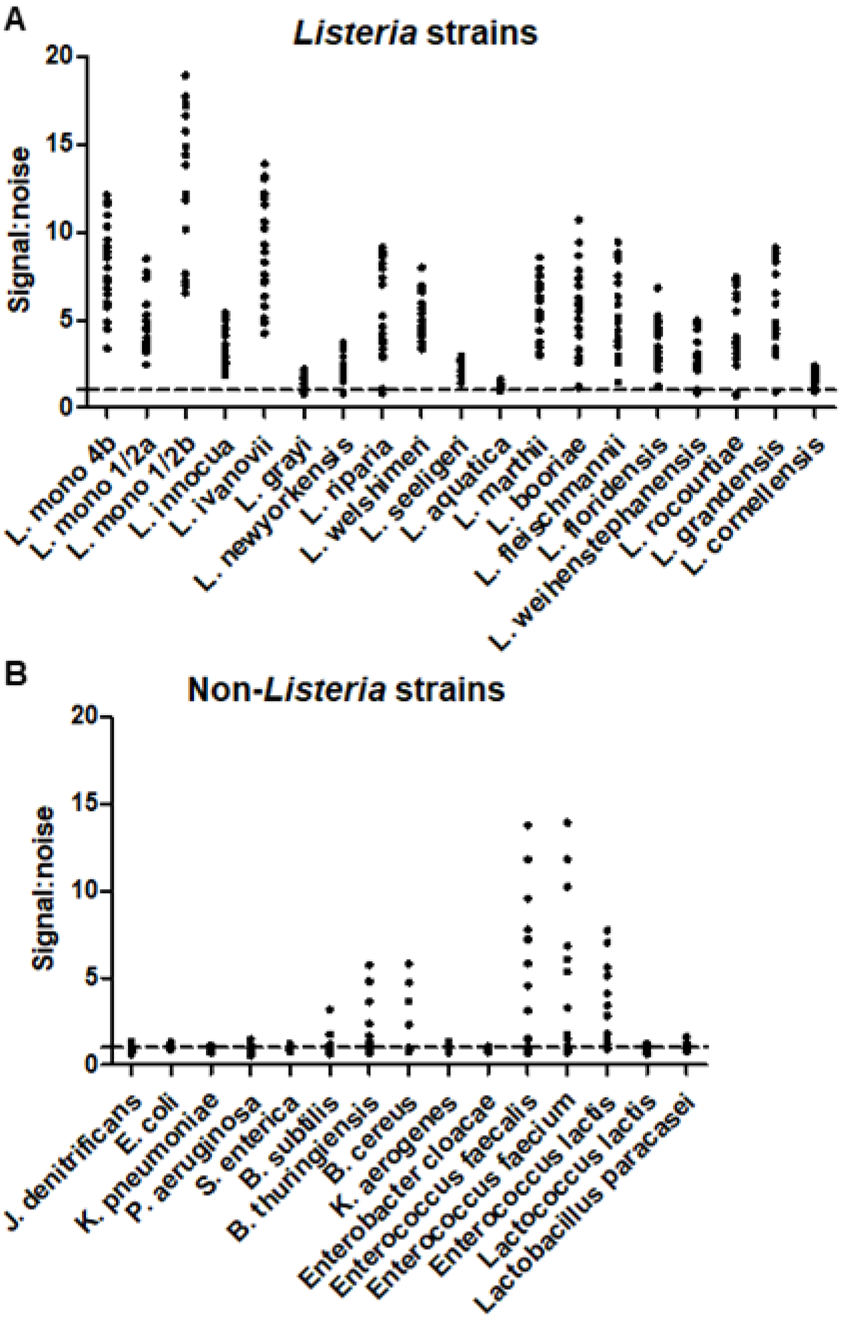

Figure S4. The signal-to-noise ratio of the 20 antibodies targeting PDC-E2 plotted for each strain. (**A**) The 19 *Listeria* strains were employed to test the sensitivity of the test. Among them, it is important to observe that the species *L. grayi*, *L. aquatica*, and *L. cornellensis* were the ones with the lowest signal:noise ratio, being slightly above the value of 1 (dashed line). (**B**) The 15 non-*Listeria* strains were used to determine the specificity of the test. Most of them showed low signal:noise ratio, being basically on the value of 1 (dashed line). Interestingly, the species from genus *Bacillus* and *Enterococcus* were the only ones presenting considerable reaction with some antibodies, which may indicate that PDC-E2 is also accessible on the surface of the cells, and that the protein shows considerable similarity to that one of *Listeria*. Each point of the graphic represents one of the antibodies, which were tested against every strain identified in the x-axis.

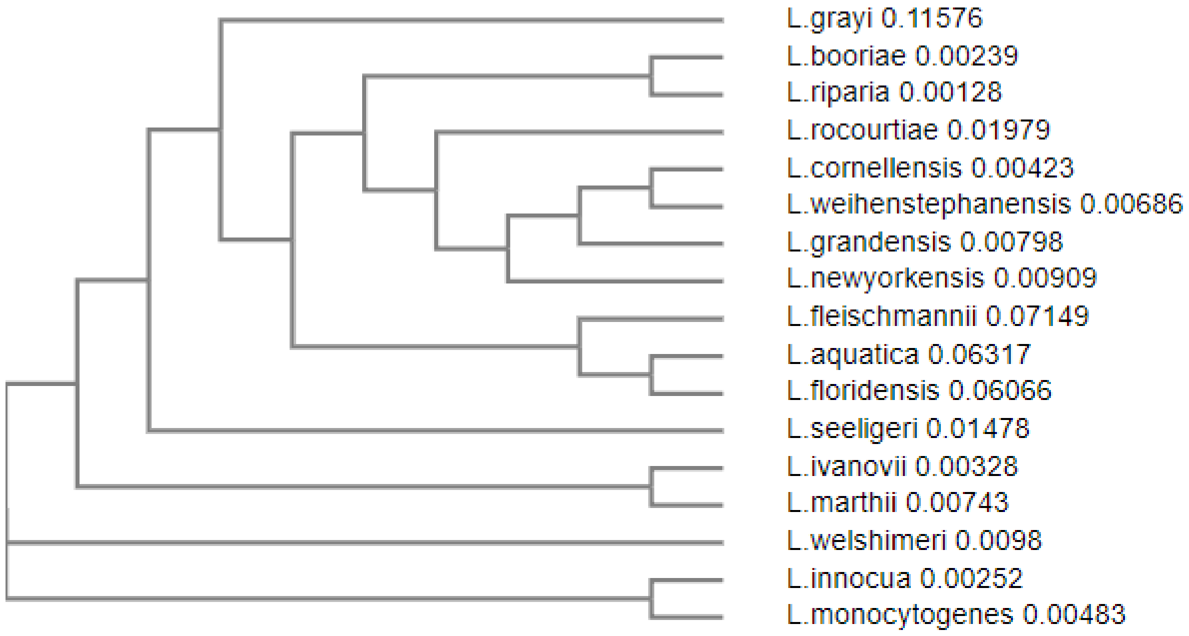

Figure S5. Phylogenetic tree of the *Listeria* spp. used in indirect ELISA based on the PDC-E2 protein sequence. The GenBank protein codes are described in the S1 Table. The numbers after the species name represent the level of genetic change.

A

		130-H1	133-A4	133-E2	134-C1	313-B1	313-B12	313-C9	313-D6	313-D10	313-E1	313-E9	313-F5	313-F7	313-G5	313-G10	313-G11	313-G12	313-H8	313-H11	313-F10
Listeria monocytogenes	4b	10,320	9,888	8,952	11,736	12,000	8,904	5,976	8,472	11,616	8,832	11,760	7,200	12,552	9,312	10,320	8,544	7,392	10,968	3,384	4,488
	1/2a	7,385	4,923	3,385	7,000	8,000	5,885	3,154	4,654	7,385	3,615	7,423	4,923	8,769	6,500	6,269	5,269	4,500	7,731	3,615	3,731
	1/2b	17,216	16,330	13,261	18,034	19,466	14,898	6,545	13,159	17,727	13,841	17,284	10,159	19,364	14,455	17,420	14,386	12,170	15,750	7,023	7,602
L. innocua		4,519	4,416	3,221	4,857	5,377	4,130	2,234	3,610	5,221	4,182	4,234	2,909	5,558	4,494	4,234	3,558	3,039	5,013	1,844	2,052
L. ivanovii		13,194	11,403	10,005	13,544	13,981	10,223	4,238	8,869	13,063	10,617	11,927	7,165	13,850	10,529	12,364	9,306	8,301	12,189	4,850	5,112
L. grayi		1,721	0,931	1,721	1,862	1,918	1,044	0,790	0,649	1,072	1,698	1,975	0,931	2,144	1,636	2,229	1,693	1,777	2,003	1,100	1,552
L. newyorkensis		3,442	3,395	3,112	4,244	4,031	0,802	1,532	2,405	3,631	3,418	3,395	2,334	4,102	3,395	0,754	1,580	1,768	3,725	1,886	2,523
L. riparia		8,235	8,975	8,034	10,723	9,748	0,840	3,361	5,479	8,773	8,639	7,395	5,244	10,118	8,000	0,908	3,025	3,664	8,941	3,899	4,639
L. welshimeri		6,620	6,892	5,713	7,526	7,496	5,622	3,718	4,897	6,620	5,924	6,650	4,987	7,587	5,955	5,804	5,380	4,262	6,952	4,413	3,446
L. seeligeri		2,605	2,634	2,239	3,512	3,015	2,327	1,551	2,034	2,649	2,429	2,751	2,166	3,000	2,415	2,576	2,093	1,932	2,810	1,785	1,405
L. aquatica		1,317	1,185	1,295	1,471	1,559	1,207	0,988	1,120	1,361	1,207	1,580	1,251	1,515	1,185	1,580	1,361	1,098	1,624	0,966	1,295
L. marthii		6,821	7,362	5,886	8,111	8,007	6,302	3,744	5,511	7,529	6,738	7,009	5,490	8,589	6,738	6,655	6,114	5,220	7,092	3,036	3,494
L. booriae		7,375	6,083	6,750	8,917	9,000	1,208	3,333	5,917	10,708	6,958	9,417	4,125	9,083	6,583	1,292	3,333	5,042	8,667	2,583	2,833
L. fleischmannii		7,500	4,944	6,333	7,944	8,944	5,056	3,833	5,500	8,833	6,333	9,444	1,444	10,333	6,611	9,000	7,111	4,389	8,444	2,944	3,500
L. floridensis		4,190	5,157	4,289	5,926	6,000	1,240	3,223	4,116	4,959	4,537	4,240	4,339	6,868	3,595	4,983	3,124	3,471	4,934	2,653	2,901
L. weihenstephanensis		4,498	4,685	4,413	5,465	5,380	1,018	2,325	3,479	4,939	4,719	4,803	2,580	5,686	4,803	1,188	2,240	2,818	4,973	2,190	2,563
L. rocourtiae		6,984	8,000	7,027	8,670	7,632	0,778	4,043	4,670	7,027	7,330	6,227	3,589	7,849	6,422	0,800	2,789	3,611	7,178	3,070	3,805
L. grandensis		8,329	8,703	8,227	9,824	9,688	0,884	4,589	6,119	9,110	8,737	7,615	5,949	9,756	8,431	1,326	3,399	4,589	8,805	3,093	4,215
L. cornellensis		2,298	2,225	2,043	2,845	2,663	0,948	1,204	1,350	2,261	2,043	1,970	1,568	2,298	2,043	1,021	1,058	1,131	2,371	1,204	2,006
Jonesia denitrificans		0,708	0,708	0,761	0,735	1,601	0,761	0,866	1,181	0,971	1,050	0,971	0,630	0,682	0,761	1,128	1,338	0,945	1,076	0,971	0,892
Escherichia coli		1,045	1,098	1,152	1,205	1,259	1,125	1,071	1,152	1,179	1,152	1,071	0,857	0,857	0,804	0,884	1,045	0,991	1,098	1,045	1,286
Klebsiella pneumoniae		0,970	0,736	0,937	0,770	1,506	1,138	0,770	0,803	0,970	1,004	1,004	0,836	1,037	0,736	0,870	0,970	0,937	1,004	1,004	1,037
Pseudomonas aeruginosa		0,719	0,758	0,894	[illegible]	0,739	0,661	0,622	0,778	1,050	0,855	0,836	0,583	0,855	0,855	0,972	1,089	0,952	1,477	0,933	1,166
Salmonella enterica		0,903	0,961	1,107	0,728	1,165	1,078	0,903	0,961	1,165	0,961	0,757	0,903	0,816	0,786	0,961	0,903	0,990	1,223	0,903	1,165
Bacillus subtilis		0,922	1,118	1,431	0,647	1,098	1,196	1,020	1,118	0,941	1,196	0,922	0,725	3,275	1,333	3,490	0,863	0,824	0,863	0,882	0,961
B. thuringiensis		0,846	1,057	3,836	0,634	1,087	0,846	0,725	1,057	0,876	0,785	1,087	0,695	5,799	1,419	2,748	1,691	0,785	1,178	3,624	4,802
B. cereus		0,834	0,949	4,083	0,748	1,035	0,834	0,748	0,834	1,006	0,891	0,863	0,748	5,118	1,466	2,070	0,978	0,891	0,949	3,652	5,808
Klebsiella aerogenes		0,946	1,089	0,917	0,831	1,089	1,204	0,745	0,860	1,032	0,946	1,003	0,975	0,831	0,831	1,118	1,232	1,232	0,975	0,860	1,376
Enterobacter cloacae		0,911	1,021	1,049	0,801	1,187	0,773	0,856	0,856	0,966	0,773	0,966	0,856	0,911	0,939	0,828	0,911	0,856	1,021	1,077	1,021
Enterococcus faecalis		0,753	7,938	11,182	11,994	8,845	0,927	0,715	0,888	0,869	11,800	0,657	0,985	15,721	1,352	14,099	1,506	0,830	0,908	0,811	7,783
Enterococcus faecium		1,480	8,160	17,000	11,920	9,680	0,920	0,840	1,120	1,000	11,800	1,080	0,720	14,600	2,120	14,120	1,760	1,080	1,480	1,000	5,360
Enterococcus lactis		1,814	3,148	7,357	6,673	6,947	0,992	0,958	1,232	1,369	7,049	1,266	0,890	8,281	2,087	8,281	1,471	0,958	1,163	1,198	2,875
Lactococcus lactis		0,989	1,088	1,434	0,841	1,137	0,989	1,088	0,890	1,088	1,137	0,989	0,841	0,890	0,989	1,088	1,236	0,742	0,890	1,187	0,841
Lactobacillus paracasei		0,932	1,291	1,865	0,968	1,865	0,968	0,932	1,076	1,183	1,255	1,147	0,753	0,968	0,968	1,147	1,219	1,614	1,112	0,968	1,219

B

		130-H1	133-A4	133-E2	134-C1	313-B1	313-B12	313-C9	313-D6	313-D10	313-E1	313-E9	313-F5	313-F7	313-G5	313-G10	313-G11	313-G12	313-H8	313-H11	313-F10
Listeria monocytogenes	4b	9,576	9,576	8,688	10,704	10,872	8,232	4,392	6,792	10,464	7,800	11,088	6,816	12,120	7,296	8,328	6,720	6,744	8,808	4,032	3,984
	1/2a	6,154	4,423	2,923	5,962	6,615	5,038	2,269	3,731	6,346	3,154	6,923	4,731	8,500	5,346	5,385	4,462	4,000	5,846	2,923	3,423
	1/2b	14,898	16,636	11,352	16,159	16,432	13,057	3,477	10,193	15,852	13,091	15,648	7,057	18,920	11,011	12,682	12,136	11,148	13,023	7,091	6,750
L. innocua		3,896	4,649	2,909	4,260	4,545	3,506	1,429	2,909	4,701	3,610	3,896	2,883	5,403	3,792	3,558	3,065	2,857	4,104	1,948	2,416
L. ivanovii		11,272	11,578	8,738	11,228	12,146	9,393	2,752	7,602	11,184	9,306	12,015	5,636	13,893	8,563	10,180	7,646	7,602	9,393	4,893	4,456
L. grayi		1,326	1,016	1,524	1,749	1,467	0,734	0,677	0,959	0,734	1,693	1,693	0,734	2,201	1,100	2,060	1,382	1,298	2,116	0,959	1,411
L. newyorkensis		2,806	3,301	2,947	3,631	3,395	0,849	1,179	2,004	2,923	3,183	2,523	1,650	3,583	2,806	0,849	1,155	1,415	2,947	1,862	2,263
L. riparia		6,588	9,143	6,420	9,042	7,798	0,807	1,950	3,765	5,983	7,261	4,303	3,025	7,966	5,580	1,042	1,647	2,050	6,286	3,832	4,437
L. welshimeri		5,622	6,922	4,957	6,680	6,348	5,078	2,388	4,050	5,955	5,743	6,076	4,897	8,010	5,199	4,897	4,050	4,020	5,652	2,932	3,144
L. seeligeri		2,312	2,707	2,180	2,941	2,488	2,137	1,083	1,844	2,517	2,239	2,605	1,932	2,971	2,210	2,180	1,815	1,859	2,371	1,493	1,537
L. aquatica		1,185	1,163	1,361	1,537	1,339	1,405	1,098	1,076	1,295	1,273	1,339	1,098	1,427	1,032	1,471	1,163	1,010	1,229	1,054	1,163
L. marthii		5,657	7,965	4,596	7,549	6,842	5,761	2,267	4,388	6,614	6,198	6,260	5,220	8,568	5,532	5,490	5,241	4,659	6,239	3,203	3,161
L. booriae		6,417	6,250	6,708	8,292	7,875	1,042	2,333	4,542	8,708	6,458	7,583	2,375	7,833	4,542	1,000	1,667	2,792	6,375	2,708	2,292
L. fleischmannii		4,722	4,944	5,944	7,889	7,000	4,833	3,222	3,944	7,333	5,500	8,167	1,000	8,611	3,778	7,667	4,000	2,556	6,556	2,778	2,889
L. floridensis		3,223	5,231	3,868	5,355	4,959	0,868	2,058	3,421	3,942	4,264	3,967	3,967	6,818	3,322	3,992	2,281	3,595	3,843	2,504	2,851
L. weihenstephanensis		3,632	4,515	3,751	4,668	4,311	0,730	1,562	2,902	3,938	4,515	3,751	1,629	4,803	3,649	0,917	1,511	1,986	3,819	2,274	2,444
L. rocourtiae		5,016	7,438	5,470	7,286	5,989	0,627	2,595	3,697	5,232	6,811	4,281	2,314	6,486	4,670	0,757	1,730	2,335	5,038	2,897	3,286
L. grandensis		6,459	8,839	6,323	8,601	7,683	0,884	2,448	4,521	6,799	8,499	5,949	3,739	8,567	5,881	0,918	1,768	2,788	6,697	2,958	3,739
L. cornellensis		1,641	1,751	1,678	2,261	2,079	0,985	0,802	1,021	1,568	1,496	1,204	1,787	2,188	1,860	0,839	0,839	1,021	1,678	1,386	2,079
Jonesia denitrificans		0,682	0,787	0,866	0,813	0,971	1,050	0,866	0,971	0,945	1,391	1,050	0,708	0,840	0,840	0,892	1,207	1,102	0,945	0,866	0,813
Escherichia coli		1,045	0,884	1,152	0,991	1,045	1,232	0,884	0,938	0,938	0,884	1,098	0,804	1,018	1,045	0,830	0,911	0,991	1,098	0,991	0,964
Klebsiella pneumoniae		0,770	0,970	0,836	0,796	0,970	0,770	0,903	0,970	0,836	0,803	0,870	0,736	1,037	0,937	1,071	0,937	1,037	0,870	0,987	1,271
Pseudomonas aeruginosa		0,680	0,719	0,700	0,583	0,719	0,739	0,641	0,700	0,778	0,719	0,661	0,564	0,797	0,739	0,836	0,836	0,914	1,283	0,933	1,108
Salmonella enterica		0,816	0,903	1,223	0,903	1,078	0,961	0,786	1,136	1,049	1,194	1,223	0,874	0,816	0,990	0,961	0,874	0,990	1,311	1,019	0,932
Bacillus subtilis		1,039	0,784	1,314	0,725	1,059	1,176	0,804	0,804	0,980	0,882	0,745	0,667	3,176	0,843	2,510	1,196	0,824	0,961	1,137	0,882
B. thuringiensis		0,846	1,057	3,111	0,695	0,815	0,785	0,846	0,876	1,087	0,755	0,966	0,664	5,738	0,846	1,359	0,906	1,057	0,997	3,745	4,893
B. cereus		0,805	0,805	2,962	0,891	1,064	0,805	0,776	0,863	0,834	0,891	0,776	0,805	4,744	0,748	1,553	0,978	1,006	0,863	4,141	5,780
Klebsiella aerogenes		1,003	0,889	1,146	0,774	0,889	0,946	0,717	1,032	0,717	0,917	0,860	1,003	0,745	0,688	1,118	0,889	1,061	0,917	0,688	1,290
Enterobacter cloacae		0,911	0,994	0,828	0,773	0,718	0,801	1,160	0,828	0,828	1,021	1,021	0,690	0,801	0,745	0,663	0,911	0,663	1,104	1,077	1,021
Enterococcus faecalis		0,927	7,223	10,796	8,768	6,200	0,946	0,618	0,811	0,753	11,376	0,657	0,869	13,770	0,850	10,661	1,082	0,753	0,888	0,946	7,494
Enterococcus faecium		1,600	6,080	13,320	9,880	5,800	1,000	0,880	1,080	1,080	10,760	0,960	0,920	13,920	0,920	11,600	1,120	1,200	1,280	1,040	5,160
Enterococcus lactis		1,198	3,422	6,844	6,091	5,030	1,095	0,924	1,061	1,369	7,221	2,430	0,856	7,734	1,095	7,255	0,821	0,411	1,061	1,848	2,909
Lactococcus lactis		0,890	1,038	1,286	0,841	0,989	1,137	0,841	0,989	1,088	0,791	1,038	0,692	0,890	1,187	1,038	1,137	1,038	1,187	1,335	1,038
Lactobacillus paracasei		1,040	1,112	1,865	0,861	1,255	0,896	0,717	1,183	1,147	0,896	1,076	0,610	1,076	1,040	1,112	1,183	1,291	0,861	1,040	1,040

C

		130-H1	133-A4	133-E2	134-C1	313-B1	313-B12	313-C9	313-D6	313-D10	313-E1	313-E9	313-F5	313-F7	313-G5	313-G10	313-G11	313-G12	313-H8	313-H11	313-F10
Listeria monocytogenes	4b	7,176	9,456	6,456	9,192	7,968	7,176	2,736	5,040	8,136	6,576	9,072	4,728	10,536	4,896	5,736	6,000	5,664	6,720	3,528	3,768
	1/2a	4,385	4,231	2,462	4,000	4,615	4,000	1,385	2,846	4,731	2,577	5,231	4,385	7,269	3,346	3,423	3,269	3,038	4,154	2,654	2,923
	1/2b	10,705	14,932	7,227	14,420	11,830	11,250	2,114	7,023	11,386	11,114	12,170	4,500	13,705	6,955	7,636	10,364	8,795	9,920	5,727	6,307
L. innocua		3,013	4,519	2,338	3,377	3,584	2,935	1,065	2,104	3,351	3,091	3,221	2,909	5,065	2,623	2,494	2,701	2,442	3,143	1,636	1,506
L. ivanovii		8,257	11,053	6,335	8,869	8,869	7,951	2,184	5,155	8,214	8,301	9,262	3,495	11,621	5,767	7,252	6,073	5,767	6,684	4,456	4,063
L. grayi		1,185	0,987	1,411	1,636	1,298	0,818	0,705	0,818	0,903	1,382	1,524	0,903	2,116	1,100	1,665	1,354	0,987	1,721	1,721	1,326
L. newyorkensis		1,957	3,301	2,381	2,923	2,381	0,660	0,919	1,297	2,075	2,947	1,910	1,391	3,112	1,862	0,896	0,943	1,014	2,098	1,721	2,169
L. riparia		3,429	8,605	4,168	7,025	4,605	0,874	1,076	1,950	3,462	6,958	2,891	1,815	5,546	2,857	1,076	1,109	1,345	3,429	3,966	4,269
L. welshimeri		4,202	6,892	3,385	5,924	4,685	4,383	1,451	2,872	4,504	4,866	4,987	4,564	6,529	3,778	3,325	3,506	3,446	4,353	2,388	2,932
L. seeligeri		1,829	2,737	1,683	2,678	2,151	1,976	0,834	1,376	2,078	2,005	2,327	1,771	2,824	1,800	1,639	1,683	1,668	2,268	1,478	1,332
L. aquatica		0,988	1,163	1,076	1,339	1,141	1,120	1,163	1,076	1,163	1,141	1,141	1,163	1,339	1,076	1,317	0,944	0,944	1,076	1,032	1,120
L. marthii		4,908	7,175	2,995	6,884	5,033	4,991	1,955	3,390	5,241	5,075	4,991	4,451	5,948	3,744	3,099	4,679	4,409	4,950	3,265	3,036
L. booriae		4,958	5,958	5,833	5,958	5,500	1,125	1,833	2,792	6,125	5,708	4,792	1,250	6,583	2,917	1,125	1,500	1,958	3,750	2,083	2,292
L. fleischmannii		2,833	4,611	5,167	5,889	4,889	3,389	2,556	3,389	5,722	4,778	6,389	0,944	7,556	2,556	5,889	2,778	1,444	4,667	2,667	2,722
L. floridensis		2,702	5,306	2,752	4,835	4,116	0,843	1,884	2,306	2,777	3,545	3,372	3,322	5,678	2,157	2,430	1,810	2,380	2,752	2,504	2,950
L. weihenstephanensis		2,393	4,379	2,597	3,734	3,072	0,730	1,137	1,816	2,614	3,853	2,342	1,307	3,547	2,122	0,849	1,086	1,205	2,427	2,173	2,342
L. rocourtiae		2,832	7,135	3,395	5,492	3,676	0,865	1,254	2,184	3,222	5,470	2,681	1,492	4,757	2,422	0,670	1,232	1,254	3,222	2,941	3,568
L. grandensis		3,875	8,533	4,079	6,527	4,861	0,918	1,292	2,414	3,773	6,935	3,501	2,006	6,629	2,992	0,952	1,224	1,530	3,671	2,822	3,773
L. cornellensis		1,313	1,641	1,459	2,043	1,678	1,021	0,948	0,985	1,167	1,496	1,094	1,422	1,787	1,386	1,094	1,058	0,985	1,094	1,386	2,116
Jonesia denitrificans		0,866	0,866	0,866	0,630	1,023	0,918	1,286	1,259	1,155	1,233	1,076	0,682	1,050	0,945	0,840	1,076	0,971	0,866	0,840	0,866
Escherichia coli		1,045	1,098	0,991	1,339	1,098	0,884	0,884	0,884	0,911	0,804	0,938	1,018	1,045	0,964	0,911	0,884	0,911	1,045	1,232	1,152
Klebsiella pneumoniae		0,836	0,703	1,037	1,037	0,970	0,903	0,736	1,004	0,836	0,602	1,104	0,970	1,305	0,703	0,903	1,004	1,171	1,104	0,836	1,439
Pseudomonas aeruginosa		0,661	0,661	0,952	[illegible]	0,661	0,661	0,739	0,836	0,739	0,875	0,641	0,641	0,855	0,661	0,836	0,972	0,836	0,875	0,816	0,991
Salmonella enterica		0,874	0,932	0,932	0,816	0,786	0,903	1,049	1,282	0,816	1,223	0,816	0,816	0,757	0,845	0,874	0,845	0,990	0,903	0,845	0,757
Bacillus subtilis		0,824	0,902	0,843	0,647	0,863	0,686	0,824	0,686	0,706	0,784	0,765	0,804	3,353	0,980	1,765	0,824	1,275	1,000	0,961	0,824
B. thuringiensis		0,755	1,208	2,386	0,755	0,815	0,906	0,755	0,966	0,997	0,966	1,268	0,876	5,285	1,359	1,329	1,027	1,299	1,027	3,715	4,983
B. cereus		0,690	0,719	2,329	0,834	0,834	0,748	0,834	0,690	0,891	0,690	0,776	0,920	4,687	0,748	1,006	1,006	0,805	1,035	3,824	5,866
Klebsiella aerogenes		0,917	0,917	0,946	0,803	0,717	0,717	0,917	0,774	0,774	0,889	0,774	0,975	1,089	1,003	1,032	0,889	0,889	0,917	0,803	1,232
Enterobacter cloacae		0,966	0,856	0,911	0,883	0,745	0,801	0,773	1,132	0,856	1,021	0,856	0,718	0,883	0,828	0,939	0,828	0,994	1,021	0,883	1,049
Enterococcus faecalis		0,830	5,852	9,560	4,539	3,129	0,946	0,695	0,773	0,792	9,464	0,715	0,888	7,725	1,024	5,833	0,908	0,850	0,927	1,178	7,957
Enterococcus faecium		1,200	6,080	10,240	6,080	3,280	0,800	1,080	1,360	0,840	9,440	0,840	1,000	11,480	0,800	6,840	1,040	0,960	1,120	1,920	4,600
Enterococcus lactis		1,232	2,772	5,612	4,106	2,806	1,027	1,061	1,335	0,958	5,852	1,061	0,856	7,118	1,061	5,133	0,958	1,095	0,958	0,958	2,601
Lactococcus lactis		0,989	1,236	0,989	0,643	0,989	0,890	0,890	0,890	0,791	0,940	0,841	0,742	0,890	0,841	0,890	0,791	0,940	1,088	1,038	0,940
Lactobacillus paracasei		0,968	1,434	1,004	0,789	1,040	1,040	0,896	0,968	0,968	1,183	1,004	0,825	1,076	1,040	1,147	1,255	1,542	0,896	2,080	1,112

Figure S6. The signal-to-noise ratio of the scFv-Fc tested in indirect ELISA. Three different concentrations were used: (**A**) EC_{50}+; (**B**) EC_{50}; and (**C**) EC_{50}-. The color scale goes from green (high reaction) to red (low reaction) going through white (signal-to-noise ratio = 1).

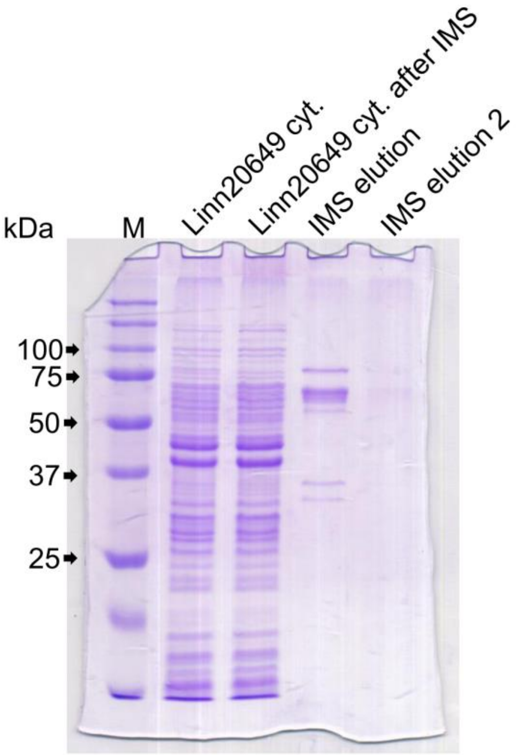

Figure S7. Complete figure of the SDS-PAGE after IMS for target identification. Of note, only the first 4 wells were included in Fig. 2A of the article.

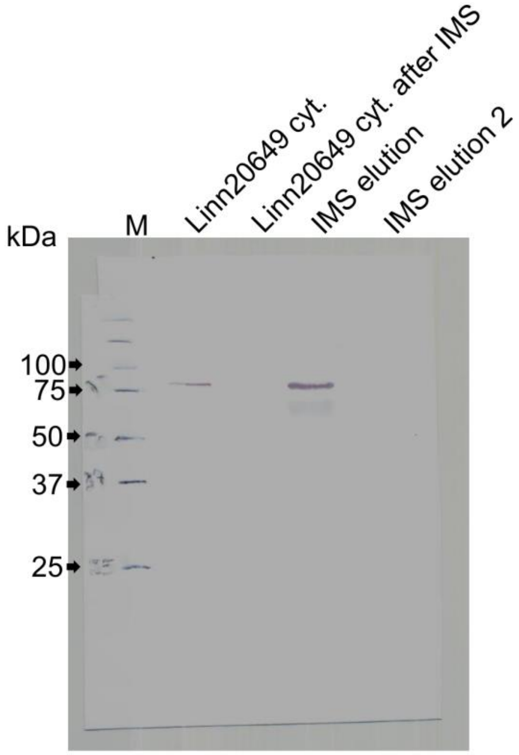

Figure S8. Complete figure of the immunoblot with GSM133-A4 after IMS for target identification. Of note, only the 4th well (IMS elution) was included in Fig. 2A of the article.

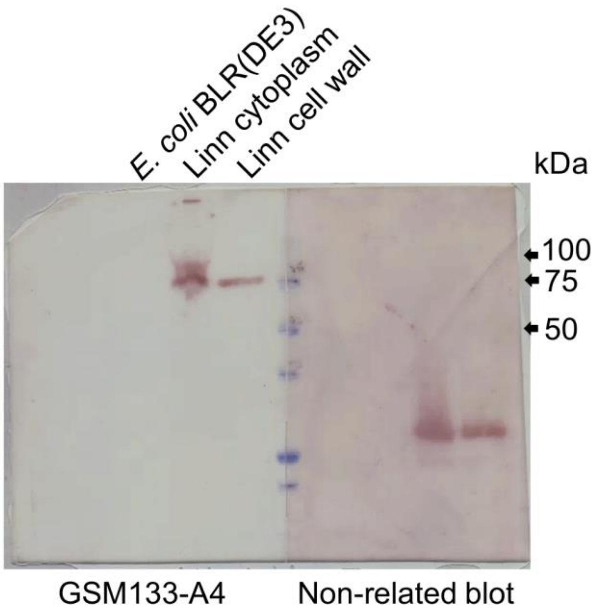

Figure S9. Complete figure of the immunoblot with GSM133-A4 to determine PDC-E2 cellular location. Of note, only the labeled wells were included in Fig. 3A of the article.

Text S1. Resulting sequences from ORFeome panning used to determine the MSR of the 4 initial antibodies.

```
GSM259-C2       ------------------------------------------------------------   0
GSM259-A12      ------------------------------------------------------------   0
GSM259-D11      ------------------------------------------------------------   0
GSM259-A1       ------------------------------------------------PVSGTIKEIKVA   12
GSM259-D6       YSFKLPDIGEGIHEGEIVKWFVQPGDKIEEDESLFEVQNDKSVEEITSPVSGTIKEIKVA   60
GSM259-F12      ------------------------------------------------------------   0


GSM259-C2       --------------------------------------------------FEFKLPDIGE   10
GSM259-A12      ------------------------------PKAESTESTPAPAQASGKGIFEFKLPDIGE   30
GSM259-D11      -------------------------------KAESTESTPAPAQASGKGIFEFKLPDIGE   29
GSM259-A1       EGTVATVGQVLVTFDGVEGHEDDAEEESAAPKAESTESTPAPAQASGKGIFEFKLPDIGE   72
GSM259-D6       EGTVATVGQVLVTFDGVEGHEDDAEEESAAPKAESTESTPAPAQASGKGIFEFKLPDIGE   120
GSM259-F12      -----------------------------------TESTPAPAQASGKGIFEFKLPDIGE   25
                                                                  **********

GSM259-C2       GIHEGEIVKWFIQPGDKVEEDQSIFEVQNDKSVEEITSPVDGTVKDILVSEGTVATVGQV   70
GSM259-A12      GIHEGEIVKWFIQPGDKVEEDQSIFEVQNDKSVEEITSPVDGTVKDILVSEGTVATVGQV   90
GSM259-D11      GIHEGEIVKWFIQPGDKVEEDQSIFEVQNDKSVEEITSPVDGTVKDILVSEGTVATVGQV   89
GSM259-A1       GIHEGEIVKWFIQPGDKVEEDQSIFEVQNDKSVEEITSPVDGTVKDILVSEGTVATVGQV   132
GSM259-D6       GIHEGEIVKWFIQPGDKVEEDQSIFEVQNDKSVEEITSPVDGTVKDILVSEGTVATVGQV   180
GSM259-F12      GIHEGEIVKWFIQPGDKVEEDQSIFEVQNDKSVEEITSPVDGTVKDILVSEGTVATVGQV   85
                ************************************************************

GSM259-C2       LVTFEGDFEGEASHESTPESPAEEAELTNNDATSAPVTGGNGTPSSKKDPNGL          123
GSM259-A12      LVTFEGDFEGEASHESTPESPAEEAELTNNDATSAPVTGGNGTPSSKKDPNGL          143
GSM259-D11      LVTFEGDFEGEASHESTPESPAEEAELTNNDATS-------------------          123
GSM259-A1       LVTFEGDFEGEA-----------------------------------------          144
GSM259-D6       LVTFEGDFEGEASHESTPESPAEEAELTN------------------------          209
GSM259-F12      LVTFEGDFEGEASHESTPESPAEEAEL--------------------------          112
                ************
```

MRS sequence for GSM130-H1 scFv-Fc with GSM259 sequences:
FEFKLPDIGEGIHEGEIVKWFIQPGDKVEEDQSIFEVQNDKSVEEITSPVDGTVKDILVSEGTVATVGQVLVTFEGDFEGEA

```
GSM260-E1       SFKLPDIGEGIHEGEIVKWFVQPGDKIEEDESLFEVQNDKSVEEITSPVSGTIKEIKVAE
                GTVATVGQVLVTFDGVEGHEDDAEEESAAPKAESTESTPAPAQASGKGIFEFKLPDIGEG
                IHEGEIVKWFIQPGDKVEEDQSIFEVQNDKSVEE
```

MRS sequence for GSM133-A4 scFv-Fc with GSM260 sequences: GSM260-E1 was the unique hit

```
GSM261-G1       TESTPAPAQASGKGIFEFKLPDIGEGIHEGEIVKWFIQPGDKVEEDQSIFEVQNDKSVEE
                ITSPVDGTVKDILVSEGTVATVGQVLVTFEGDFEGEASHESTPESPAEEAEL
```

MRS sequence for GSM133-E2 scFv-Fc with GSM261 sequences: GSM261-G1 was the unique hit

Text S2. Resulting sequences from single gene panning used to determine the MSR of the 4 initial antibodies.

```
SaK20-A2      -------------------AYSFKLPDIGEGIHEGEIVKWFVQPGDKIEEDESLFEVQND     41
SaK20-G4      PKAESTESTPAPAQASGKGIFEFKLPDIGEGIHEGEIVKWFVQPGDKIEEDESLFEVQND     60
SaK46-F9      -KAESTESTPAPAQASGKGIFEFKLPDIGEGIHEGEIVKWFVQPGDKIEEDESLFEVQND     59
                                  :.**************************************

SaK20-A2      KSVEEITSPVSGTIKEIKVAEGTVATVGQVLVTFDGVEGHEDDAEEESAAPKAESTESTP     101
SaK20-G4      KSVEEITSPVSGTIKEIKVAEGTVATVGQVLVTFDGVEGHE-------------------     101
SaK46-F9      KSVEEITSPVSGTIKEIKVAEGTVATVGQVLVTFDGVEGHEDDAEEESAAPKAEST----     115
              *****************************************

SaK20-A2      APAQASGKGIFEF      114
SaK20-G4      -------------      101
SaK46-F9      -------------      115
```

MRS sequence of 1st LD for GSM133-A4 scFv-Fc with SaK20 and SaK46 sequences:

FKLPDIGEGIHEGEIVKWFVQPGDKIEEDESLFEVQNDKSVEEITSPVSGTIKEIKVAEGTVATVGQVLVTFDGVEGHE

```
SaK46-H2       --------------------------PAQASGKGIFEFKLPDIGEGIHEGEIVKWFIQPG    34
SaK46-G1       GVEGHEDDAEEESAAPKAESTESTPAPAQASGKGIFEFKLPDIGEGIHEGEIVKWFIQPG    60
SaK46-E10      ------------------ESTESTPAPAQASGKGIFEFKLPDIGEGIHEGEIVKWFIQPG    42
SaK46-E6       -------------------------APAQASGKGIFEFKLPDIGEGIHEGEIVKWFIQPG    35
SaK46-D12      ------------------------PAPAQASGKGIFEFKLPDIGEGIHEGEIVKWFIQPG    36
SaK46-D9       ----------------KAESTESTPAPAQASGKGIFEFKLPDIGEGIHEGEIVKWFIQPG    44
SaK46-D3       ------------------ESTESTPAPAQASGKGIFEFKLPDIGEGIHEGEIVKWFIQPG    42
SaK46-C3       -----EDDAEEESAAPKAESTESTPAPAQASGKGIFEFKLPDIGEGIHEGEIVKWFIQPG    55
SaK46-A4       ------------------------------SGKGIFEFKLPDIGEGIHEGEIVKWFIQPG    30
SaK20-H1       ---------------PKAESTESTPAPAQASGKGIFEFKLPDIGEGIHEGEIVKWFIQPG    45
SaK20-F2       -----------------------TPAPAQASGKGIFEFKLPDIGEGIHEGEIVKWFIQPG    37
SaK20-E1       ----------------------------QASGKGIFEFKLPDIGEGIHEGEIVKWFIQPG    32
SaK20-B1       ---------------------ESTPAPAQASGKGIFEFKLPDIGEGIHEGEIVKWFIQPG    39
SaK20-B3       ---------------------ESTPAPAQASGKGIFEFKLPDIGEGIHEGEIVKWFIQPG    39
SaK20-B4       ------------------------PAPAQASGKGIFEFKLPDIGEGIHEGEIVKWFIQPG    36
SaK20-C2       ----------------------------QASGKGIFEFKLPDIGEGIHEGEIVKWFIQPG    32
SaK46-C6       --------------------------PAQASGKGIFEFKLPDIGEGIHEGEIVKWFIQPG    34
SaK46-F5       ------------------------------SGKGIFEFKLPDIGEGIHEGEIVKWFIQPG    30
                                             ******************************

SaK46-H2       DKVEEDQSIFEVQNDKSVEEITSPVDGTVKDILVSEGTVATVGQVLVTFEGDFEGEASHE    94
SaK46-G1       DKVEEDQSIFEVQNDKSVEEITSPVDGTVKDILVSEGTVATVGQVLVTFEGDFEGEASHE    120
SaK46-E10      DKVEEDQSIFEVQNDKSVEEITSPVDGTVKDILVSEGTVATVGQVLVTFEGDFEGEASHE    102
SaK46-E6       DKVEEDQSIFEVQNDKSVEEITSPVDGTVKDILVSEGTVATVGQVLVTFEGDFEGEASHE    95
SaK46-D12      DKVEEDQSIFEVQNDKSVEEITSPVDGTVKDILVSEGTVATVGQVLVTFEGDFEGEASHE    96
SaK46-D9       DKVEEDQSIFEVQNDKSVEEITSPVDGTVKDILVSEGTVATVGQVLVTFEGDFEGEASHE    104
SaK46-D3       DKVEEDQSIFEVQNDKSVEEITSPVDGTVKDILVSEGTVATVGQVLVTFEGDFEGEASHE    102
SaK46-C3       DKVEEDQSIFEVQNDKSVEEITSPVDGTVKDILVSEGTVATVGQVLVTFEGDFEGEASHE    115
SaK46-A4       DKVEEDQSIFEVQNDKSVEEITSPVDGTVKDILVSEGTVATVGQVLVTFEGDFEGEASHE    90
SaK20-H1       DKVEEDQSIFEVQNDKSVEEITSPVDGTVKDILVSEGTVATVGQVLVTFEGDFEGEASHE    105
SaK20-F2       DKVEEDQSIFEVQNDKSVEEITSPVDGTVKDILVSEGTVATVGQVLVTFEGDFEGEAS--    95
SaK20-E1       DKVEEDQSIFEVQNDKSVEEITSPVDGTVKDILVSEGTVATVGQVLVTFEGDFEGEASHE    92
SaK20-B1       DKVEEDQSIFEVQNDKSVEEITSPVDGTVKDILVSEGTVATVGQVLVTFEGDFEGEASHE    99
SaK20-B3       DKVEEDQSIFEVQNDKSVEEITSPVDGTVKDILVSEGTVATVGQVLVTFEGDFEGEASHE    99
SaK20-B4       DKVEEDQSIFEVQNDKSVEEITSPVDGTVKDILVSEGTVATVGQVLVTFEGDFEGEASHE    96
SaK20-C2       DKVEEDQSIFEVQNDKSVEEITSPVDGTVKDILVSEGTVATVGQVLVTFEGDFEGEASHE    92
SaK46-C6       DKVEEDQSIFEVQNDKSVEEITSPVDGTVKDILVSEGTVATVGQVLVTFEGDFEGEASHE    94
SaK46-F5       DKVEEDQSIFEVQNDKSVEEITSPVDGTVKDILVSEGTVATVGQVLVTFEGDFEGEASHE    90
               **********************************************************

SaK46-H2       STPESPAEEAELTNNDATSAPVTGGNGTPSSKKDPNGLV---- 133
SaK46-G1       STPESPAEEAELTNNDATSAPVTGGNGTPSSKKDPNGLV---- 159
SaK46-E10      STPESPAEEAELTNNDATSAPVTG------------------- 126
SaK46-E6       STPESPAEEAELTNNDATSAPVTGGNGTPSSK----------- 127
SaK46-D12      STPESPAEEAELTNNDATSAPVTGG----------------- 122
```

```
SaK46-D9       STPESPAEE---------------------------------- 113
SaK46-D3       STPESPAEEAELTNNDATSAPVTGGNGTPSSKKDPNGL----- 140
SaK46-C3       STP---------------------------------------- 118
SaK46-A4       STPESPAEEAELT------------------------------ 103
SaK20-H1       STPESPAEEAELTNNDATSAPVTGGNGT--------------- 133
SaK20-F2       ------------------------------------------- 95
SaK20-E1       STPESPAEEAELTNNDATSAPVTGGNGTPSSKKDP-------- 127
SaK20-B1       STPESPAEEAELTNNDATSAPVTGGNGTPSSKKDPNGLVIAMP 142
SaK20-B3       STPESPAEEAELTNNDATSAPVTGGNGTPSSKKDPNGLVIA-- 140
SaK20-B4       STPESPAEEAELTNNDATSAPVTGGNGT--------------- 124
SaK20-C2       STPESPA------------------------------------ 99
SaK46-C6       STPESPA------------------------------------ 101
SaK46-F5       S------------------------------------------ 91
```

MRS sequence of 2nd LD for GSM133-A4 scFv-Fc with SaK20 and SaK46 sequences:

SGKGIFEFKLPDIGEGIHEGEIVKWFIQPGDKVEEDQSIFEVQNDKSVEEITSPVDGTVKDILVSEGTVATVGQVLVTFEGDFEGEAS

```
SaK48-B6       ------------------------------------------TESTPAPAQASGKGIFEF   18
SaK48-G9       -------------------TFDGVEGHEDDAEEESAAPKAESTESTPAPAQASGKGIFEF   41
SaK48-E11      -------------------TFDGVEGHEDDAEEESAAPKAESTESTPAPAQASGKGIFEF   41
SaK20-E11      ------------------------------------APKAESTESTPAPAQASGKGIFEF   24
SaK48-D7       IKEIKVAEGTVATVGQVLVTFDGVEGHEDDAEEESAAPKAESTESTPAPAQASGKGIFEF   60
SaK48-G10      --------------------------------------KAESTESTPAPAQASGKGIFEF   22
SaK48-G2       -----------------------------------------------APAQASGKGIFEF   13
SaK48-F12      --------------------------------------KAESTESTPAPAQASGKGIFEF   22
SaK48-D9       ------------------------------------------TESTPAPAQASGKGIFEF   18
SaK48-B11      ----------------------------------------------PAPAQASGKGIFEF   14
SaK20-B11      -------------------------------------------ESTPAPAQASGKGIFEF   17
SaK48-B7       --------------------------------------------------QASGKGIFEF   10
SaK48-E4       ----------------------------------------------PAPAQASGKGIFEF   14
SaK48-E9       ----------------------------------------------PAPAQASGKGIFEF   14
                                                                 **********

SaK48-B6       KLPDIGEGIHEGEIVKWFIQPGDKVEEDQSIFEVQNDKSVEEITSPVDGTVKDILVSEGT   78
SaK48-G9       KLPDIGEGIHEGEIVKWFIQPGDKVEEDQSIFEVQNDKSVEEITSPVDGTVKDILVSEGT   101
SaK48-E11      KLPDIGEGIHEGEIVKWFIQPGDKVEEDQSIFEVQNDKSVEEITSPVDGTVKDILVSEGT   101
SaK20-E11      KLPDIGEGIHEGEIVKWFIQPGDKVEEDQSIFEVQNDKSVEEITSPVDGTVKDILVSEGT   84
SaK48-D7       KLPDIGEGIHEGEIVKWFIQPGDKVEEDQSIFEVQNDKSVEEITSPVDGTVKDILVSEGT   120
SaK48-G10      KLPDIGEGIHEGEIVKWFIQPGDKVEEDQSIFEVQNDKSVEEITSPVDGTVKDILVSEGT   82
SaK48-G2       KLPDIGEGIHEGEIVKWFIQPGDKVEEDQSIFEVQNDKSVEEITSPVDGTVKDILVSEGT   73
SaK48-F12      KLPDIGEGIHEGEIVKWFIQPGDKVEEDQSIFEVQNDKSVEEITSPVDGTVKDILVSEGT   82
SaK48-D9       KLPDIGEGIHEGEIVKWFIQPGDKVEEDQSIFEVQNDKSVEEITSPVDGTVKDILVSEGT   78
SaK48-B11      KLPDIGEGIHEGEIVKWFIQPGDKVEEDQSIFEVQNDKSVEEITSPVDGTVKDILVSEGT   74
SaK20-B11      KLPDIGEGIHEGEIVKWFIQPGDKVEEDQSIFEVQNDKSVEEITSPVDGTVKDILVSEGT   77
SaK48-B7       KLPDIGEGIHEGEIVKWFIQPGDKVEEDQSIFEVQNDKSVEEITSPVDGTVKDILVSEGT   70
SaK48-E4       KLPDIGEGIHEGEIVKWFIQPGDKVEEDQSIFEVQNDKSVEEITSPVDGTVKDILVSEGT   74
SaK48-E9       KLPDIGEGIHEGEIVKWFIQPGDKVEEDQSIFEVQNDKSVEEITSPVDGTVKDILVSEGT   74
               ************************************************************

SaK48-B6       VATVGQVLVTFDGVEGHEDDAEEE------------------------------------   102
SaK48-G9       VATVGQVLVTFEGDFEGEASHESTPESPAEEAELTNNDATSAPVTGG-------------   148
SaK48-E11      VATVGQVLVTFEGDFEGEASHESTPESPAEEA----------------------------   133
SaK20-E11      VATVGQVLVTFEGDFEG-------------------------------------------   101
SaK48-D7       VATVGQVLVTFEGDFEG-------------------------------------------   137
SaK48-G10      VATVGQVLVTFEGDFEGEASHESTPESPAEEAELTNN-----------------------   119
SaK48-G2       VATVGQVLVTFEGDFEGEASHESTPESPAEEAELTNNDATSAPVTGGNGTPSSKKDPNG-   132
SaK48-F12      VATVGQVLVTFEGDFEGEASHESTPESPAEEAELTNN-----------------------   119
SaK48-D9       VATVGQVLVTFEGDFEGEASHESTPES---------------------------------   105
SaK48-B11      VATVGQVLVTFEGDFEGEASHESTPESPAEEAELTNNDATSAPVTGGNGTPSSKKDPNGL   134
SaK20-B11      VATVGQVLVTFEGDFEGEASHESTPESPAEEAELTNNDATSAPVTGGNG-----------   126
SaK48-B7       VATVGQVLVTFEGDFEGEA-----------------------------------------   89
SaK48-E4       VATVGQVLVTFEGDFEGEASHESTPESPAEEAELTNNDATSAPVTG--------------   120
SaK48-E9       VATVGQVLVTFEGDFEGEAS----------------------------------------   94
               ***********:*
```

```
SaK48-B6       -   102
SaK48-G9       -   148
SaK48-E11      -   133
SaK20-E11      -   101
SaK48-D7       -   137
SaK48-G10      -   119
SaK48-G2       -   132
SaK48-F12      -   119
SaK48-D9       -   105
SaK48-B11      V   135
SaK20-B11      -   126
SaK48-B7       -   89
SaK48-E4       -   120
SaK48-E9       -   94
```

MRS sequence for GSM134-C1 scFv-Fc with SaK20 and SaK48 sequences:
QASGKGIFEFKLPDIGEGIHEGEIVKWFIQPGDKVEEDQSIFEVQNDKSVEEITSPVDGTVKDILVSEGTVATVGQVLVTFEGDFEG

```
SaK50-H1      -------NNQL---HQQLLLK----------------------QKKKQQHQ--------K  20
SaK20-D6      LRDFPVLNTTLDDATEELVYKHYFNVGIAADTDHGLYVPVIKNADKKSVFQISDEINELA  60
SaK50-F1      --------------------KHYFNVGIAADTDHGLYVPVIK------------------  22
SaK20-B6      --------------------------------------------------KISLTVPSTG  10
SaK20-D7      ------------------------------------------------------------  0
SaK20-A7      ------------------------------------------------------------  0
SaK20-A9      ------------------------------------------------------------  0
SaK20-E6      ------------------------------------------------------------  0
SaK20-F6      ------------------------------------------------------------  0
SaK20-F7      ------------------------------------------------------------  0
SaK50-H8      ------------------------------------------------------------  0
SaK20-E7      ------------------------ASTTTAQTEEKAAAPKAEKAAAKQPVASSDAYPETR  36
SaK20-G6      ------------------------ASTTTAQTEEKAAAPKAEKAAAKQPVASSDAYPETR  36
SaK20-B8      ------------------------------------------------------------  0
SaK20-C6      ---------------------------------------------------SSDAYPETR  9
SaK20-C7      ---------------------------------------------------SSDAYPETR  9
```

```
SaK50-H1      QKKQQQNN-----------QLQAPML--------------------------- 35
SaK20-D6      GKA------------------RDGKLTA------------------------- 70
SaK50-F1      ----------------------------------------------------- 22
SaK20-B6      EVISSTDL-----------SFCTSKM--------------------------- 25
SaK20-D7      ----------QKAMNNIKRLLNDPELLLMEVAAA------------------- 24
SaK20-A7      ----------------MAYSFKLPELLLMEVAAA------------------- 18
SaK20-A9      -----------------AYSFKLPELLLMEVAAA------------------- 17
SaK20-E6      ----------------MAYSFKLPELLLMEVAAA------------------- 18
SaK20-F6      ----------------MAYSFKLPELLLMEVAAA------------------- 18
SaK20-F7      ----------------MAYSFKLPELLLMEVAAA------------------- 18
SaK50-H8      ----------------------CGAVC---------------------FEFTI 10
SaK20-E7      EKLTPTRRAIAKAMVNS--KHTAPHVTLMDEIEVTALMAHRKR---------- 77
SaK20-G6      EKLTPTRRAIAKAMVNS--KHTAPHVTLMDEIEVTALMAHRKR---------- 77
SaK20-B8      ----------------S--KHTAPHVTLMDEIEVTALMAHRKRFKEVAAEKGI 35
SaK20-C6      EKLTPTRRAIAKAMVNS--KHTAPHVTLMDEIEVTALMAHRKRFKEVAAEKG- 59
SaK20-C7      EKLTPTRRAIAKAMVNS--KHTAPHVTLMDEIEVTALMAHRKRFKEVAAEKG- 59
```

MRS sequence for GSM133-E2 scFv-Fc with SaK20 and SaK50 sequences: multiple sequences

```
SaK49-E6       ----------------GVEGHEDXAXXESAAPKAESTESTPAPAQASGKGIFEFKLPDIG 44
SaK49-A8       ----------------------------------------------------EFKLPDIG 8
SaK49-E4       ---------------------------------------TPAPAQASGKGIFEFKLPDIG 21
SaK49-G10      ---------------------------------------TPAPAQASGKGIFEFKLPDIG 21
SaK49-E8       AEGTVATVGQVLVTFDGVEGHEDDAEEESAAPKAESTESTPAPAQASGKGIFEFKLPDIG 60
SaK49-C1       --------------------------------------------QASGKGIFEFKLPDIG 16
```

```
SaK49-D1      --------------------------------------------QASGKGIFEFKLPDIG 16
SaK49-E2      --------------------------------------------QASGKGIFEFKLPDIG 16
SaK49-E3      ------------------------------------TESTPAPAQASGKGIFEFKLPDIG 24
SaK49-G9      ------------------------------------TESTPAPAQASGKGIFEFKLPDIG 24
                                                                  ********

SaK49-E6      EGIHEGEIVKWFIQPGDKIEEDES------------------------------------ 68
SaK49-A8      EGIHEGEIVKWFIQPGDKVEEDQSIFEVQNDKSVEEITSPVDGTVKDILVSEGTVATVGQ 68
SaK49-E4      EGIHEGEIVKWFIQPGDKVEEDQSIFEVQNDKSVEEITSPVDGTVKDILVSEGTVATVGQ 81
SaK49-G10     EGIHEGEIVKWFIQPGDKVEEDQSIFEVQNDKSVEEITSPVDGTVKDILVSEGTVATVGQ 81
SaK49-E8      EGIHEGEIVKWFIQPGDKVEEDQSIFEVQNDKSVEEITSPVDGTVKDILVSEGTVATVGQ 120
SaK49-C1      EGIHEGEIVKWFIQPGDKVEEDQSIFEVQNDKSVEEITSPVDGTVKDILVSEGTVATVGQ 76
SaK49-D1      EGIHEGEIVKWFIQPGDKVEEDQSIFEVQNDKSVEEITSPVDGTVKDILVSEGTVATVGQ 76
SaK49-E2      EGIHEGEIVKWFIQPGDKVEEDQSIFEVQNDKSVEEITSPVDGTVKDILVSEGTVATVGQ 76
SaK49-E3      EGIHEGEIVKWFIQPGDKVEEDQSIFEVQNDKSVEEITSPVDGTVKDILVSEGTVATVGQ 84
SaK49-G9      EGIHEGEIVKWFIQPGDKVEEDQSIFEVQNDKSVEEITSPVDGTVKDILVSEGTVATVGQ 84
              ******************:***:*

SaK49-E6      ---------------------------------------------------  68
SaK49-A8      VLVTFEGDFEGEASHESTPESPAEEAELTNNDATSAPVTGGNGTPSSK---  116
SaK49-E4      VLVTFEGDFEGEASHESTPESPAEEAELTNNDATSAPVTGGNGTPSSKKDP  132
SaK49-G10     VLVTFEGDFEGEASHES----------------------------------  98
SaK49-E8      VLVTFEGDFEGEASHESTPESPAEEAELTNNDATSAPVTGG---------  161
SaK49-C1      VLVTFEGDFEGEASHESTPESPAEEAELTNNDATSAPVTG----------  116
SaK49-D1      VLVTFEGDFEGEASHESTPESPAEEAELTNN--------------------  107
SaK49-E2      VLVTFEGDFEGEA--------------------------------------  89
SaK49-E3      VLVTFEGDFEGEASHESTPESPAEEAELTNNDATSAPVTGGNGTPS-----  130
SaK49-G9      VLVTFEGDFEGEASHESTPESPAEEAELTNNDATSAPVTGGNGT-------  128
```

MRS sequence of 2nd LD for GSM133-E2 IgG with SaK20 and SaK49 sequences:

EFKLPDIGEGIHEGEIVKWFIQPGDKVEEDQS

```
SaK52-F9      ------------------------------------------------------------   0
SaK52-E12     ------------------------------------------------------------   0
SaK52-E7      ------------------------------------------------------------   0
SaK52-E3      ------------------------------------------------------------   0
SaK52-C11     ------------------------------------------------------------   0
SaK52-C5      ------------------------------------------------------------   0
SaK52-B9      ------------------------------------------------------------   0
SaK52-B8      ------------------------------------------------------------   0
SaK52-B3      ------------------------------------------------------------   0
SaK20-G8      ------------------------------------------------------------   0
SaK20-G4      ------------------------------------------------------------   0
SaK20-F4      ------------------------------------------------------------   0
SaK20-D8      ------------------------------------------------------------   0
SaK20-B5      ------------------------------------------------------------   0
SaK20-A4      ------------------------------------------------------------   0
SaK20-A5      ------------------------------------------------------------   0
SaK20-C5      ------------------------------------------------------------   0
SaK20-C8      ------------------------------------------------------------   0
SaK20-D4      ------------------------------------------------------------   0
SaK20-E8      AYSFKLPDIGEGIHEGEIVKWFVQPGDKIEEDESLFEVQNDKSVEEITSPVSGTIKEIKV   60
SaK52-B11     ------------------------------------------------------------   0
SaK52-C6      ------------------------------------------------------------   0
SaK52-D3      ------------------------------------------------------------   0

SaK52-F9      ----------------GVEGHEDDAEEESAAPKAESTESTPAPAQASGKGIFEFKLPDIG   44
SaK52-E12     ----------------------------------------PAPAQASGKGIFEFKLPDIG   20
SaK52-E7      -------------------------EEESAAPKAESTESTPAPAQASGKGIFEFKLPDIG   35
SaK52-E3      ----------------------------------------------------EFKLPDIG   8
SaK52-C11     -----------------------------------------APAQASGKGIFEFKLPDIG   19
SaK52-C5      --------------------------------KAESTESTPAPAQASGKGIFEFKLPDIG   28
SaK52-B9      -------------------------------PKAESTESTPAPAQASGKGIFEFKLPDIG   29
```

```
SaK52-B8      --------------------------------------------QASGKGIFEFKLPDIG   16
SaK52-B3      -------------------------------------------AQASGKGIFEFKLPDIG   17
SaK20-G8      -------------------------------PKAESTESTPAPAQASGKGIFEFKLPDIG   29
SaK20-G4      ------------------------------APKAESTESTPAPAQASGKGIFEFKLPDIG   30
SaK20-F4      --------------------------------------------QASGKGIFEFKLPDIG   16
SaK20-D8      ---------------------TAQTEEKAAAPKAESTESTPAPAQASGKGIFEFKLPDIG   39
SaK20-B5      ----------------------------------------PAPAQASGKGIFEFKLPDIG   20
SaK20-A4      ------------------------------------TESTPAPAQASGKGIFEFKLPDIG   24
SaK20-A5      ------------------------------------TESTPAPAQASGKGIFEFKLPDIG   24
SaK20-C5      -------VGQVLVTFDGVEGHEDDAEEESAAPKAESTESTPAPAQASGKGIFEFKLPDIG   53
SaK20-C8      ------------------------------------------PAQASGKGIFEFKLPDIG   18
SaK20-D4      ------------------------------------------PAQASGKGIFEFKLPDIG   18
SaK20-E8      AEGTVATVGQVLVTFDGVEGHEDDAEEESAAPKAESTESTPAPAQASGKGIFEFKLPDIG   120
SaK52-B11     -------------------------------------ESTPAPAQASGKGIFEFKLPDIG   23
SaK52-C6      --------------FDGVEGHEDDAEEESAAPKAESTESTPAPAQASGKGIFEFKLPDIG   46
SaK52-D3      --------------------------------------------QASGKGIFEFKLPDIG   16
                                                                  ********

SaK52-F9      EGIHEGEIVKWFIQPGDKVEEDQSIFEVQNDKSVEEITSPVDGTVKDILVSEGTVATVGQ   104
SaK52-E12     EGIHEGEIVKWFIQPGDKVEEDQSIFEVQNDKSVEEITSPVDGTVKDILVSEGTVATVGQ   80
SaK52-E7      EGIHEGEIVKWFIQPGDKVEEDQSIFEVQNDKSVEEITSPVDGTVKDILVSEGTVATVGQ   95
SaK52-E3      EGIHEGEIVKWFIQPGDKVEEDQSIFEVQNDKSVEEITSPVDGTVKDILVSEGTVATVGQ   68
SaK52-C11     EGIHEGEIVKWFIQPGDKVEEDQSIFEVQNDKSVEEITSPVDGTVKDILVSEGTVATVGQ   79
SaK52-C5      EGIHEGEIVKWFIQPGDKVEEDQSIFEVQNDKSVEEITSPVDGTVKDILVSEGTVATVGQ   88
SaK52-B9      EGIHEGEIVKWFIQPGDKVEEDQSIFEVQNDKSVEEITSPVDGTVKDILVSEGTVATVGQ   89
SaK52-B8      EGIHEGEIVKWFIQPGDKVEEDQSIFEVQNDKSVEEITSPVDGTVKDILVSEGTVATVGQ   76
SaK52-B3      EGIHEGEIVKWFIQPGDKVEEDQSIFEVQNDKSVEEITSPVDGTVKDILVSEGTVATVGQ   77
SaK20-G8      EGIHEGEIVKWFIQPGDKVEEDQSIFEVQNDKSVEEITSPVDGTVKDILVSEGTVATVGQ   89
SaK20-G4      EGIHEGEIVKWFIQPGDKVEEDQSIFEVQNDKSVEEITSPVDGTVKDILVSEGTVATVGQ   90
SaK20-F4      EGIHEGEIVKWFIQPGDKVEEDQSIFEVQNDKSVEEITSPVDGTVKDILVSEGTVATVGQ   76
SaK20-D8      EGIHEGEIVKWFIQPGDKVEEDQSIFEVQNDKSVEEITSPVDGTVKDILVSEGTVATVGQ   99
SaK20-B5      EGIHEGEIVKWFIQPGDKVEEDQSIFEVQNDKSVEEITSPVDGTVKDILVSEGTVATVGQ   80
SaK20-A4      EGIHEGEIVKWFIQPGDKVEEDQSIFEVQNDKSVEEITSPVDGTVKDILVSEGTVATVGQ   84
SaK20-A5      EGIHEGEIVKWFIQPGDKVEEDQSIFEVQNDKSVEEITSPVDGTVKDILVSEGTVATVGQ   84
SaK20-C5      EGIHEGEIVKWFIQPGDKVEEDQSIFEVQNDKSVEEITSPVDGTVKDILVSEGTVATVGQ   113
SaK20-C8      EGIHEGEIVKWFIQPGDKVEEDQSIFEVQNDKSVEEITSPVDGTVKDILVSEGTVATVGQ   78
SaK20-D4      EGIHEGEIVKWFIQPGDKVEEDQSIFEVQNDKSVEEITSPVDGTVKDILVSEGTVATVGQ   78
SaK20-E8      EGIHEGEIVKWFIQPGDKVEEDQSIFEVQNDKSVEEITSPVDGTVKDILVSEGTVATVGQ   180
SaK52-B11     EGIHEGEIVKWFIQPGDKVEEDQSIFEVQNDKSVEEITSPVDGTVKDILVSEGTVATVGQ   83
SaK52-C6      EGIHEGEIVKWFIQPGDKVEEDQSIFEVQNDKSVEEITSPVDGTVKDILVSEGTVATVGQ   106
SaK52-D3      EGIHEGEIVKWFIQPGDKVEEDQSIFEVQNDKSVEEITSPVDGTVKDILVSEGTVATVGQ   76
              ************************************************************

SaK52-F9      VLVTFEGDFEGEASHESTPESP-----------------------------      126
SaK52-E12     VLVTFEGDFEGEASHESTPESPAEEAELTNNDATSAPVTGG---------      121
SaK52-E7      VLVTFEGDFEGEASHESTPESPAEEAELTNN--------------------      126
SaK52-E3      VLVTFEGDFEGEASHES----------------------------------      85
SaK52-C11     VLVTFEGDFEGEASHESTPESPAEEAELTNNDATSAPVTGGNGTPSSK---      127
SaK52-C5      VLVTFEGDFEGEASHESTPESPAEEAEL-----------------------      116
SaK52-B9      VLVTFEGDFEGEASHESTPESPAEEAELTNNDATSAPVTGG---------      130
SaK52-B8      VLVTFEGDFEGEA--------------------------------------      89
SaK52-B3      VLVTFEGDFEGEASHESTPESPAE---------------------------      101
SaK20-G8      VLVTFEGDFEGEASHESTPESPAEEAELTNNDATSAPVTGGNGTPSS----      136
SaK20-G4      VLVTFEGDFEGEA--------------------------------------      103
SaK20-F4      VLVTFEGDFEGEA--------------------------------------      89
SaK20-D8      VLVTFEGDFEGEASHESTPESPA----------------------------      122
SaK20-B5      VLVTFEGDFEGEASHESTPESPAEEAELTNNDATSAPVTGGNGTPSSKKDP      131
SaK20-A4      VLVTFEGDFEGEASHESTPESPAE---------------------------      108
SaK20-A5      VLVTFEGDFEGEASHESTPESPAE---------------------------      108
SaK20-C5      VLVTFEGDFEGEASHESTPESPAEEA-------------------------      139
SaK20-C8      VLVTFEGDFEG----------------------------------------      89
SaK20-D4      VLVTFEGDFEG----------------------------------------      89
SaK20-E8      VLVTFEGDFEGEASHESTPESPAEEAELTNNDATSAPVTGGNGTPSSK---      228
SaK52-B11     VLVTFEGDFEGEASHESTP--------------------------------      102
SaK52-C6      VLVTFEGDFEGEASHESTPESPAEEA-------------------------      132
SaK52-D3      VLVTFEG--------------------------------------------      83
```

****

MRS sequence for GSM130-H1 scFv-Fc with SaK20 and SaK52 sequences:

EFKLPDIGEGIHEGEIVKWFIQPGDKVEEDQSIFEVQNDKSVEEITSPVDGTVKDILVSEGTVATVGQVLVTFEG
